# A coarse-to-fine modulation classification scheme utilizing Transformers and Liquid Neural Networks for Smart Grids

**DOI:** 10.1371/journal.pone.0359526

**Published:** 2026-10-26

**Authors:** FeiFei Hu, Yu Huang, Xubin Lin, Guanhuan Huang

**Affiliations:** 1 Power Dispatching and Control Center, China Southern Power Grid (CSG), Guangzhou, Guangdong, China; 2 College of Electronics and Information Technology, South China University of Technology, Guangzhou, Guangdong, China; Hubei University, CHINA

## Abstract

Dependable data transmission is vital for modern Smart Grid operations, facilitating essential services like demand-side management and Advanced Metering Infrastructure (AMI). Within these networks, Automatic Modulation Recognition (AMR) acts as a crucial technique for blind signal processing and system security. However, the highly volatile nature of Power Line Communication (PLC) channels severely restricts AMR performance. Traditional statistical algorithms generally fail under severe noise conditions (low SNR), whereas standard single-stage deep learning architectures lack transparency and struggle to distinguish highly similar modulation schemes. To overcome these limitations, we introduce an innovative two-tier recognition architecture that integrates statistical feature engineering with cutting-edge neural networks. The preliminary stage executes coarse classification by feeding Higher-Order Cumulants (HOCs) into a Transformer module, effectively categorizing signals into general modulation families (e.g., PSK or QAM). Here, the Transformer’s self-attention mechanism excels at identifying complex correlations among statistical parameters. Subsequently, based on the preliminary result, a specialized Liquid Neural Network (LNN) is deployed for fine-grained analysis. By processing 3D constellation matrices, these specialized LNN models pinpoint the exact modulation order. Their inherent continuous-time properties provide improved adaptability to the temporal fluctuations and heavy noise typical of PLC environments, as evidenced by our low-SNR evaluations. Experimental evaluations confirm that the introduced hierarchical strategy yields markedly higher accuracy rates than traditional algorithms and unified deep learning baseline models, especially in high-noise scenarios. Ultimately, this structured design delivers a highly transparent and robust solution for AMR in challenging communication setups.

## 1. Introduction

The 21st-century power system is undergoing a profound and necessary transformation from a centralized, passive grid to a decentralized, intelligent Smart Grid [1,2]. This evolution, driven by the integration of digital technology into every facet of the energy lifecycle, is revolutionizing how we generate, distribute, and consume electricity, promising paradigm-shifting enhancements in operational efficiency, grid stability, and environmental sustainability [3,4]. At the very heart of this new paradigm lies a sophisticated communication infrastructure, the grid’s “digital nervous system,” which must reliably support a vast and heterogeneous ecosystem of mission-critical applications. These applications range from the fine-grained, massive data collection of Advanced Metering Infrastructure (AMI) to the real-time, low-latency control required for dynamic load balancing and the seamless coordination of distributed energy resources (DERs) like solar panels and electric vehicles [5]. Among the various communication modalities, Power Line Communication (PLC) presents a uniquely compelling proposition, leveraging the existing electrical wiring as a data transmission medium, thereby offering a cost-effective and near-ubiquitous communication backbone that avoids the need for new infrastructure deployment [6,7].

Despite its logistical advantages, the PLC channel is notoriously inhospitable and presents a formidable environment for reliable communication. It is characterized by a litany of impairments, including frequency-selective fading due to multipath propagation, significant distance-dependent path loss, and colored background noise. Most pernicious, however, is the prevalence of non-Gaussian, impulsive noise, which emanates from the high-frequency switching operations of countless electronic appliances connected to the grid [8,9]. This impulsive noise introduces large, sporadic disturbances that can severely corrupt transmitted signals, overwhelm conventional receivers, and cripple communication systems that rely on Gaussian noise assumptions.

In this complex and dynamic setting, Automatic Modulation Recognition (AMR) emerges as an indispensable enabling technology for intelligent and autonomous receiver operations [10,11]. As a core component of physical-layer signal intelligence and cognitive radio, AMR empowers a device to sense and identify the modulation scheme of a received signal without prior arrangement with the transmitter. This capability is instrumental for several key Smart Grid functions, fostering a more resilient and efficient communication network. It promotes interoperability by allowing devices from different vendors to communicate seamlessly; it facilitates dynamic spectrum management by enabling devices to intelligently identify and utilize vacant frequency bands; it enhances network security by serving as a physical-layer intrusion detection mechanism capable of flagging anomalous signals; and it enables performance optimization by allowing a receiver to adapt its demodulation and decoding algorithms to match the incoming signal, thereby maximizing data throughput [12–14]. Ultimately, robust AMR is the bedrock upon which higher-level AI applications for grid intelligence are built. The efficacy of AI-driven predictive control and real-time analytics [15] is entirely contingent on the fidelity of the data they ingest, as intermittent data loss from AMR failures can destabilize these advanced systems. Therefore, ensuring the integrity of this foundational data link is a direct and necessary contribution to making the future AI-enabled smart grid more robust, resilient, and achievable”.

Historically, AMR research has followed two main trajectories. The first, likelihood-based (LB) methods, are statistically optimal under specific assumptions but suffer from prohibitive computational complexity and an impractical requirement for precise a priori knowledge of channel and noise statistics, which is seldom available in PLC networks [16,17]. The second, feature-based (FB) methods, offer a more pragmatic alternative by extracting handcrafted features–-such as Higher-Order Cumulants (HOCs) or cyclostationary features–-and classifying them using decision trees or simple thresholds [18,19]. While computationally efficient and interpretable, the discriminative power of these features erodes rapidly at low signal-to-noise ratios (SNRs), and their performance is often brittle, hinging on meticulously tuned thresholds that lack adaptability to time-varying channel conditions.

The recent ascendancy of deep learning (DL) has ushered in a new era for AMR. End-to-end models, particularly Convolutional Neural Networks (CNNs) that treat signals as images and Recurrent Neural Networks (RNNs) that process them as time series, have demonstrated a powerful ability to learn features automatically, often outperforming traditional methods [20,21]. However, these monolithic models are not a panacea. Their “black-box” nature obscures the decision-making process, hindering interpretability and trust. More critically, they struggle with the challenge of intra-class similarity, frequently confusing spectrally adjacent schemes like 16-QAM and 64-QAM. This “all-at-once” classification approach is structurally naive, as it fails to capture the nuanced hierarchies inherent in modulation families, treating the classification of BPSK versus QPSK as a problem of equivalent difficulty to 16-QAM versus 64-QAM. While some initial hybrid models have attempted to combine DL with features, they typically do so through simple concatenation, failing to address this fundamental structural limitation [22,23].

While recent advancements in Automatic Modulation Recognition (AMR) have increasingly leveraged deep learning to bypass manual feature engineering [24], their direct application to Power Line Communication (PLC) networks remains highly problematic. Recent studies have made significant strides in applying advanced deep learning to automated feature extraction [25], robust environmental measurement and anti-interference techniques [26], and complex time-series classification using novel neural network architectures [27]. However, despite these cutting-edge developments, the PLC physical layer is notoriously afflicted by severe, non-Gaussian impulsive noise, which routinely overwhelms conventional discrete-time architectures such as standard CNNs and RNNs [28]. To mitigate this noise sensitivity, Higher-Order Cumulants (HOCs) have been actively utilized as robust, Gaussian-blind statistical features; however, traditional machine learning classifiers fail to fully exploit the complex, latent non-linear inter-dependencies within multidimensional HOC arrays. Although Transformer networks have recently demonstrated exceptional capability in capturing global feature correlations via self-attention mechanisms in wireless AMR tasks [29], their discrete token-processing nature fundamentally limits their adaptability to the continuous-time, transient signal anomalies (e.g., asynchronous impulsive spikes) inherent to PLC environments. Consequently, there is a critical, unaddressed need to integrate continuous-time temporal models into the PLC-AMR pipeline. Liquid Neural Networks (LNNs), governed by dynamic Ordinary Differential Equations (ODEs), offer a compelling architectural solution by fluidly adjusting their integration time-constants to “absorb” and recover from unexpected impulsive spikes [30]. Therefore, synergizing the statistical robustness of HOCs, the global relational capacity of Transformers, and the continuous-time resilience of LNNs represents a necessary evolutionary step for achieving highly reliable AMR in hostile PLC channels.

To bridge this critical gap and explicitly differentiate our work from the aforementioned advanced literature [25–27], we propose a novel Hierarchical Two-Stage Framework, termed H-TLNN(Hierarchical Transformer-Liquid Neural Network), as illustrated in Fig 1. Compared to these generalized deep learning approaches, our framework’s unique novelty lies in combining the strict statistical robustness of Higher-Order Cumulants (HOCs) with the continuous-time, adaptive dynamics of Liquid Neural Networks (LNNs). This architectural synergy provides a highly targeted, specialized solution uniquely capable of dynamically ‘absorbing’ and mitigating the severe non-Gaussian impulsive noise endemic to PLC channels. This integrated architecture, termed ‘Statistically-Guided Deep Temporal Analysis’, provides the following specific contributions:

**Fig 1:**
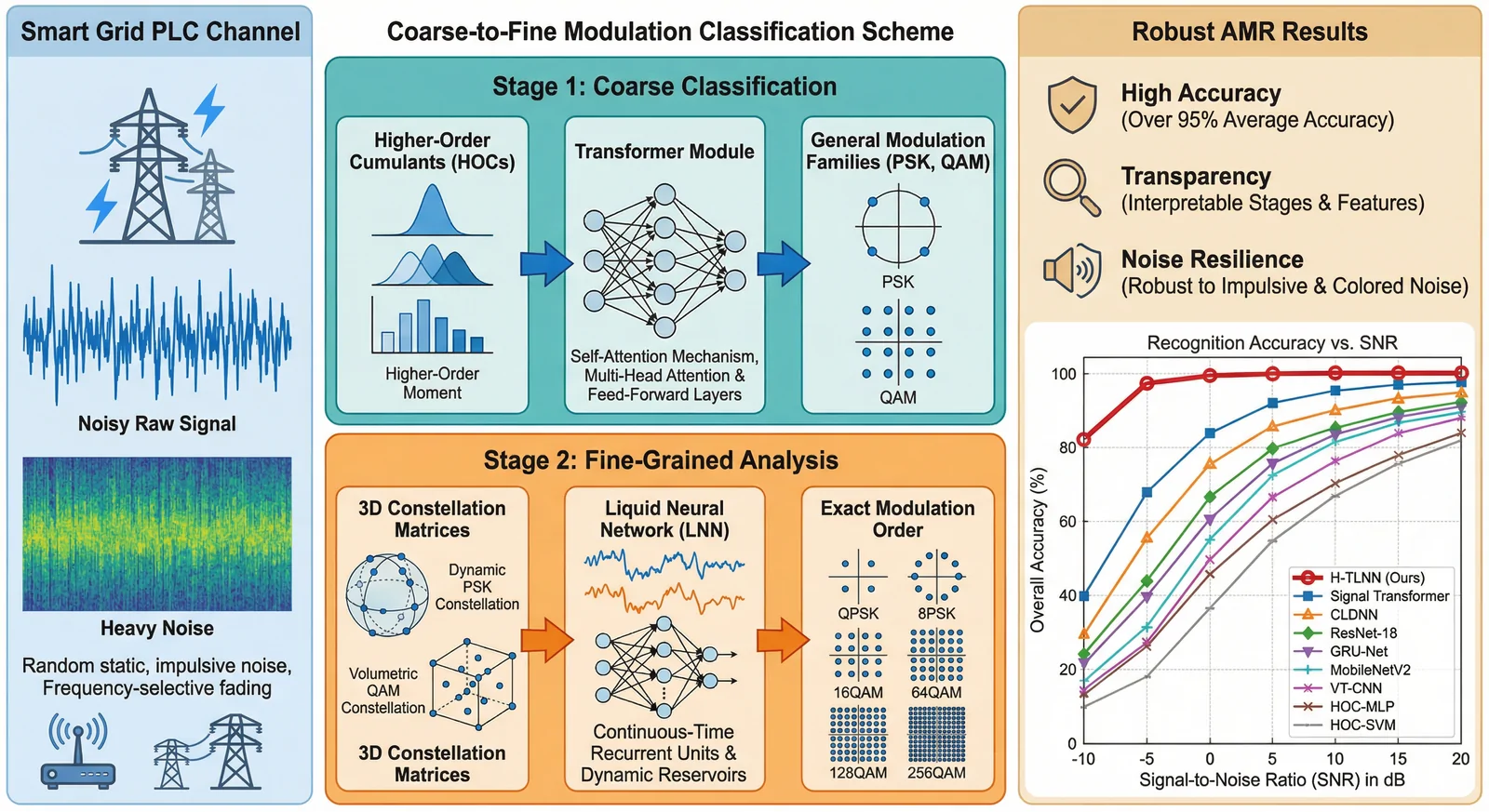
The illustration of the robust modulation recognition in smart grid communications.

- We propose a customized integration of a Transformer network with Higher-Order Cumulants (HOCs) for coarse family classification. This specific combination leverages the self-attention mechanism to capture latent non-linear dependencies among statistical features, providing highly robust, Gaussian-noise-resistant performance.
- We introduce a specialized pairing of Liquid Neural Networks (LNNs) with time-evolving 3D constellation matrices for the fine-grained recognition stage. We demonstrate that the inherent continuous-time dynamics of LNNs are systematically better suited to “absorb” the transient, high-energy impulsive noise typical of PLC channels compared to traditional discrete-time architectures.
- We establish that the synergistic integration of these two stages–-using statistical guidance to route signals to continuous-time temporal experts–-effectively decomposes the complex PLC modulation recognition task. Through comprehensive experiments, we confirm that this specific architectural integration yields significantly higher accuracy and noise resilience under multipath fading and impulsive noise than monolithic end-to-end models.

The remainder of this paper is organized as follows. Section 2 provides a detailed mathematical description of the proposed H-TLNN framework, including the feature extraction and classifier design for each stage. Section 3 outlines the experimental setup, including the dataset generation, channel modeling, and benchmark models used for comparison. Section 4 presents and discusses the comprehensive simulation results, analyzing the performance of our framework and validating its superiority. Finally, Section 5 concludes the paper and suggests avenues for future research.

## 2. System model: Indoor PLC-MIMO networks

A prerequisite for any meaningful performance evaluation of advanced communication algorithms is the establishment of a system model that possesses both mathematical rigor and high fidelity to the physical reality of the intended operational environment. Our work, therefore, is predicated on a broadband indoor Power Line Communication (PLC) network that leverages a Multiple-Input Multiple-Output (MIMO) architecture. This approach is not merely a theoretical construct but is a pragmatic solution to the channel capacity limitations of PLC systems [31,32]. It is directly motivated by the physical topology of standard three-wire indoor electrical installations, which universally comprise a Phase (P), a Neutral (N), and a Protective Earth (PE) conductor. By strategically employing these conductors to create distinct signal propagation paths–-for instance, by injecting signals in a differential mode between P-N and P-PE–-the ostensibly single-path electrical grid is transmuted into a rich, multi-channel communication medium. This effectively creates spatial diversity, providing a powerful mechanism to combat the severe frequency-selective fading endemic to PLC channels and thereby enhancing both data throughput and link reliability.

### 2.1 Channel model: Multipath and MIMO propagation

As illustrated in Fig 2, the fundamental input-output relationship governing this M×N PLC-MIMO system [33,34], under the assumption of linear time-invariance (LTI) for a static network topology, is elegantly captured in the frequency domain by the canonical linear matrix equation:


$$\begin{array}{c} Y(f)=H(f)X(f)+W(f) \end{array}$$ (1)

where $X(f)={\left[X_{1}(f),X_{2}(f),...,X_{M}(f)\right]}^{T}$ and $Y(f)={\left[Y_{1}(f),Y_{2}(f),...,Y_{M}(f)\right]}^{T}$ represent the complex vectors of the transmitted and received signals across the two modes at frequency *f*, respectively. The term W(f) denotes the additive noise vector, a complex stochastic process whose unique and challenging characteristics will be detailed subsequently. The linchpin of this model is the M×N channel transfer matrix, H(f), which holistically describes the propagation environment and the electromagnetic coupling between the constructed paths:


$$\begin{array}{c} H(f)=\left[\begin{array}{cccc} h_{1,1}(f) & h_{1,1}(f) & \cdots & h_{1,N}(f)\\ h_{2,1}(f) & h_{2,2}(f) & \cdots & h_{2,N}(f)\\ \vdots & \vdots & & \vdots \\ h_{M,1}(f) & h_{M,2}(f) & \cdots & h_{M,N}(f) \end{array}\right] \end{array}$$ (2)

**Fig 2:**
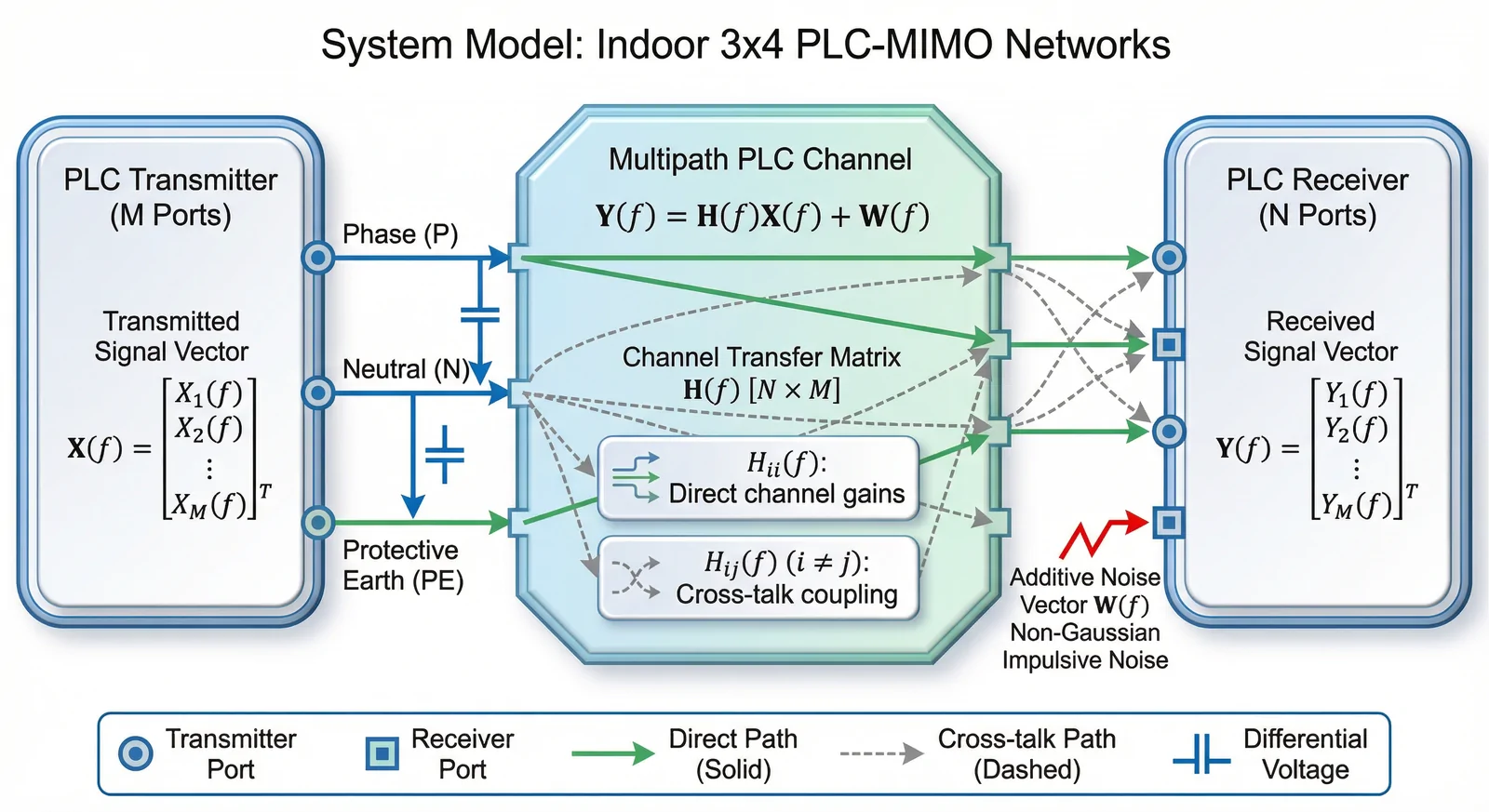
The illustration of the indoor PLC-MIMO system model.

Within this matrix, the diagonal entries, ${\;}_{ii}(f)$, represent the direct channel transfer functions for each mode. The off-diagonal entries, $H_{ij}(f)$ for i≠j, are of paramount importance as they quantify the degree of inter-modal coupling–-colloquially known as cross-talk. This cross-talk arises from the close proximity of the conductors within the power cable, leading to electromagnetic field leakage from one mode to another. The severity of this coupling is frequency-dependent and can lead to significant inter-channel interference, posing a challenge for signal recovery.

The phenomenological behavior of each entry in H(f) is, in turn, dictated by the underlying multipath propagation phenomena inherent to power lines. Due to the complex, branching topology of indoor wiring and the myriad impedance mismatches caused by connected appliances and junction boxes, the transmitted signal propagates to the receiver via numerous distinct paths. Following the widely-adopted model by Zimmermann and Dostert, the channel impulse response for any given link, *h*(*t*), can be accurately modeled as a superposition of a finite number of discre*t*e, time-delayed, and attenuated replicas of the transmitted signal:


$$\begin{array}{c} h(t)=\sum_{i=1}^{N_{f}}g_{i}\cdot \delta \left(t-\tau_{i}\right) \end{array}$$ (3)

Here, $N_{p}$ is the number of significant propagation paths, and $\tau_{i}$ is the time delay of the *i*-th path. The path coefficient $g_{i}$ is a complex-valued quantity encapsulating the cumulative attenuation and phase shift associated with that path’s specific trajectory. This coefficient is not static but exhibits a pronounced frequency dependency, which can be further modeled as $g_{i}(f)=k_{i}\cdot A\left(f,d_{i}\right)$. The term is a product of reflection and transmission factors encountered along the path, while $A\left(f,d_{i}\right)=\exp\left(-\left(a_{0}+a_{1}f^{k}\right)d_{i}\right)$ represents the cable transfer function, governed by empirically derived parameters ($a_{0},a_{1},k$) that model dielectric and skin-effect losses over distance $d_{i}$ [12]. The frequency-domain channel response is the Fourier transform of this impulse response, leading to its characteristic frequency-selective nature, marked by deep spectral nulls where destructive interference occurs.

### 2.2 Noise model: A non-Gaussian composite process

Arguably the most distinguishing and pernicious feature of the PLC channel [35,36] is the non-Gaussian character of the additive noise vector W(f). Based on extensive empirical measurements, the noise process is not a simple AWGN process but a complex composite of several distinct noise types:

(1) **Colored Background Noise:** A relatively low-power, stationary process resulting from the superposition of countless minuscule noise sources. Its power spectral density (PSD) typically exhibits a decaying trend with frequency.
(2) **Narrowband Interference:** Comprises sinusoidal components, often with harmonics, generated by the switching power supplies of electronic appliances. This manifests as deterministic, high-power interference at specific frequencies.
(3) **Impulsive Noise:** This is the dominant noise component and is itself subdivided. It includes asynchronous impulses of high power and short duration caused by transient events (e.g., switching lights on/off), and periodic impulses synchronous with the AC mains cycle, caused by devices like light dimmers and motor controllers.

Of these, the asynchronous, high-power impulsive noise poses the greatest threat to signal integrity. To mathematically abstract this behavior, we adopt the canonical Middleton Class A model. This widely validated model posits that the noise is a result of a Poisson process of impulsive events convolved with a Gaussian background. The resulting probability density function (PDF), is elegantly articulated as a countably infinite summation of weighted Gaussian density functions:


$$\begin{array}{c} f(w)=\sum_{m=0}^{\infty }\underset{\text{Poisson Weight }}{\underbrace{\frac{e^{-A}A^{m}}{m!}}}\cdot \underset{\text{Gaussian DDF }}{\underbrace{𝒩\left(w;0,\sigma_{m}^{2}\right)}} \end{array}$$ (4)

where $N\left(w;0,\sigma_{m}^{2}\right)$ is a zero-mean Gaussian PDF with variance $\sigma_{m}^{2}=\sigma_{G}^{2}\frac{m/A+\Gamma }{1+\Gamma }$. The model’s behavior is dictated by two key parameters:

- **The Impulsive Index (*A*):** This dimensionless parameter represents the product of the average number of impulsive events per second and the mean duration of an impulse. A small value of *A* (e.g., *A* < 0.1) signifies a channel with rare but potent impulsive events, making the noise highly non-Gaussian.
- **The Gaussian-to-Impulsive variance Ratio (GIR,** $\Gamma =\sigma_{G}^{2}/\sigma_{I}^{2}$**):** This parameter quantifies the power ratio between the background Gaussian component ($\sigma_{G}^{2}$) and the impulsive component ($\sigma_{I}^{2}$). A low value of Γ indicates that the total noise power is dominated by the impulsive part.

This comprehensive system model–-encapsulating frequency-selective multipath fading, inter-modal crosstalk, and, most critically, a composite noise process dominated by non-Gaussian impulsive statistics–-thus constitutes a high-fidelity representation of the operational environment [37,38]. It serves as the definitive testbed against which the robustness and efficacy of any advanced AMR algorithm for Smart Grid communications must be rigorously benchmarked.

## 3. The proposed H-TLNN framework

The H-TLNN framework is architected as a two-stage cascade that executes a “coarse-to-fine” recognition strategy, as illustrated in Fig 3. This hierarchical design intelligently decomposes the formidable 9-class classification problem into a simple 3-class coarse classification task followed by a set of simpler, more focused sub-tasks. This structure is designed to maximize accuracy, enhance interpretability, and improve robustness against channel impairments.

**Fig 3:**
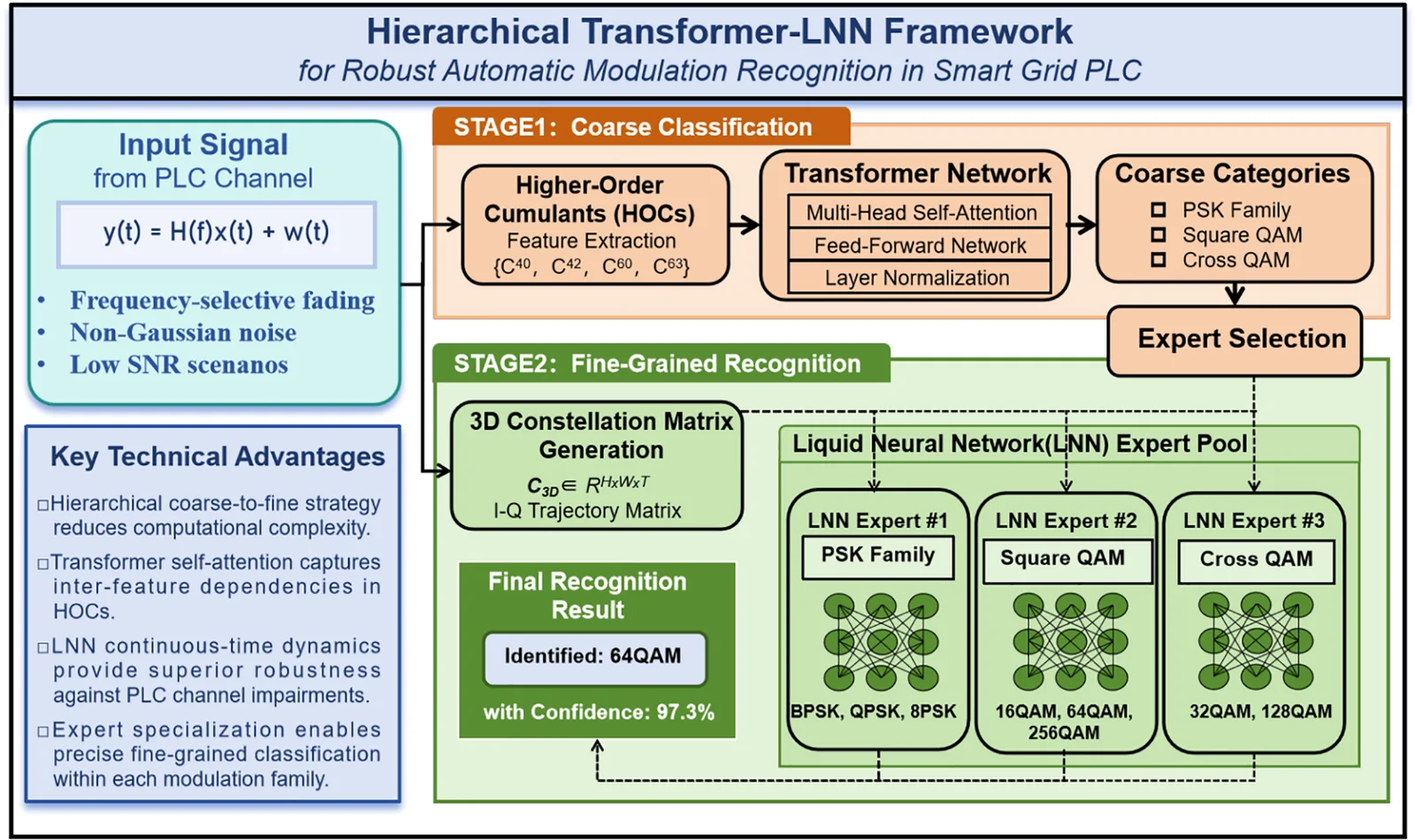
The schematic diagram of the proposed Hierarchical Transformer-LNN framework.

### 3.1 Stage 1: Transformer-based coarse classification

To effectively decompose the complex eight-class recognition problem, the proposed Stage-1 module functions as a coarse statistical router. Its explicit objective is to classify the incoming signal into one of three distinct overarching modulation families: Phase Shift Keying (PSK), Square Quadrature Amplitude Modulation (Square-QAM), and Cross Quadrature Amplitude Modulation (Cross-QAM). Specifically, the PSK family encompasses BPSK, QPSK, and 8PSK. The Square-QAM family comprises the modulations formulated on a perfect square grid, namely 16QAM, 64QAM, and 256QAM. Conversely, the Cross-QAM family includes 32QAM and 128QAM, which are characterized by their stepped, cross-like constellation boundaries. This tripartite division is fundamentally grounded in the distinct theoretical asymptotic properties of their higher-order cumulants. By deterministically separating Square-QAM from Cross-QAM at this initial stage, the hierarchical framework prevents the severe representational entanglement that typically occurs between adjacent high-order QAM schemes, thereby allowing the subsequent Stage-2 expert networks to focus exclusively on fine-grained intra-family discrimination.

#### 3.1.1 Feature extraction 1: Higher-Order Cumulants (HOCs).

The features chosen for this stage must be both computationally efficient and inherently robust to common channel distortions. Higher-Order Cumulants (HOCs) are an ideal choice, possessing several desirable properties: 1) Blindness to Gaussian Noise: By definition, all cumulants of a Gaussian process beyond the second order are zero, rendering HOCs theoretically immune to additive Gaussian noise. 2) Phase Invariance: Certain HOCs are insensitive to carrier phase and frequency offsets, obviating the need for precise carrier synchronization. 3) Distinct Signatures: Key modulation families exhibit unique theoretical HOC values. For example, for any M-PSK signal, the fourth-order cumulant *C*_42_ is non-zero, whereas for any M-QAM signal, it is zero. This provides a powerful and clear discriminant [39,40].

For a received complex baseband signal block of *L* samples, denoted as $\{r[n]{\}}_{n=1}^{L}$, where *r*[*n*] = *I*[*n*] + *jQ*[*n*], we first compute its statistical signatures. Higher-Order Cumulants (HOCs) are selected for their inherent robustness to Gaussian noise and certain channel distortions. Moreover, the (*p* + *q*)-th order cumulant of a complex random process *r* is denoted as $Cum(r[p\text{ times}],r^{*}[q\text{ times}])$, where $r^{*}$ is the complex conjugate. For a zero-mean process, the key cumulants can be expressed in terms of moments $M_{pq}=E[r^{p}(r^{*}{)}^{q}]$ as follows:


$$\begin{array}{c} C_{20}=M_{20}=E\left[r^{2}\right]\; \end{array}$$ (5)

$$\begin{array}{c} C_{21}=M_{11}=E\left[|r{|}^{2}\right]\; \end{array}$$ (6)

$$\begin{array}{c} C_{40}=M_{40}-3M_{20}^{2}\; \end{array}$$ (7)

$$\begin{array}{c} C_{41}=M_{31}-3M_{20}M_{11}\; \end{array}$$ (8)

$$\begin{array}{c} C_{42}=M_{22}-{\left|M_{20}\right|}^{2}-2M_{11}^{2} \end{array}$$ (9)

To ensure absolute fairness in comparative evaluations and to capture sufficient statistical signatures, the final feature vector fed into the Transformer comprises 12 distinct cumulants. Equation (10) is formalized as:


$$\begin{array}{c} c={\left[{\hat{C}}_{20},{\hat{C}}_{21},{\hat{C}}_{40},{\hat{C}}_{41},{\hat{C}}_{42},{\hat{C}}_{60},{\hat{C}}_{61},{\hat{C}}_{62},{\hat{C}}_{63},{\hat{C}}_{80},{\hat{C}}_{81},{\hat{C}}_{84}\right]}^{T} \end{array}$$ (10)

Furthermore, to eliminate the adverse effects of unknown channel attenuation and signal amplitude variations, a rigorous normalization procedure is applied to the raw estimated cumulants prior to network ingestion. By utilizing the signal power estimate ${\hat{C}}_{21}$ as the normalization baseline, the amplitude scaling for a cumulant of order (*p* + *q*) is defined as:


$$\begin{array}{c} {\tilde{C}}_{pq}=C_{pq}/{\left(C_{21}\right)}^{(p+q)/2} \end{array}$$ (11)

The foundation of this coarse classification stage relies on the distinct theoretical asymptotic values of these normalized cumulants across the 8 evaluated modulation families. As comprehensively summarized in Table 1, the exact 12-dimensional theoretical feature vectors exhibit mathematically proven separability. For instance, non-conjugate and asymmetric cumulants (e.g., ${\tilde{C}}_{20},{\tilde{C}}_{41},{\tilde{C}}_{61}$) effectively separate BPSK from higher-order M-PSK/M-QAM formats due to circular symmetry properties. Meanwhile, key conjugate cumulants (e.g., ${\tilde{C}}_{42},{\tilde{C}}_{63},{\tilde{C}}_{84}$) delineate clear hierarchical boundaries not only between PSK and QAM families, but also across varying QAM orders (including cross-QAM formats like 32QAM and 128QAM), providing the Stage-1 self-attention mechanism with robust, deterministic features to accurately route signals into their corresponding fine-grained expert networks.

**Table 1:** Theoretical normalized cumulant values (${\tilde{C}}_{pq}$) for all evaluated modulation families.

Cumulant	BPSK	QPSK	8PSK	16QAM	32QAM	64QAM	128QAM	256QAM
${\tilde{C}}_{20}$	1.000	0.000	0.000	0.000	0.000	0.000	0.000	0.000
${\tilde{C}}_{21}$	1.000	1.000	1.000	1.000	1.000	1.000	1.000	1.000
${\tilde{C}}_{40}$	−2.000	1.000	0.000	−0.680	−0.585	−0.619	−0.596	−0.605
${\tilde{C}}_{41}$	−2.000	0.000	0.000	0.000	0.000	0.000	0.000	0.000
${\tilde{C}}_{42}$	−2.000	−1.000	−1.000	−0.680	−0.585	−0.619	−0.596	−0.605
${\tilde{C}}_{60}$	16.000	0.000	0.000	2.080	1.812	1.797	1.745	1.697
${\tilde{C}}_{61}$	16.000	−4.000	0.000	0.000	0.000	0.000	0.000	0.000
${\tilde{C}}_{62}$	16.000	0.000	0.000	0.000	0.000	0.000	0.000	0.000
${\tilde{C}}_{63}$	16.000	4.000	4.000	2.080	1.812	1.797	1.745	1.697
${\tilde{C}}_{80}$	−272.000	−34.000	0.000	−13.980	−10.820	−11.500	−10.630	−10.510
${\tilde{C}}_{81}$	−272.000	0.000	0.000	0.000	0.000	0.000	0.000	0.000
${\tilde{C}}_{84}$	−272.000	−34.000	−34.000	−13.980	−10.820	−11.500	−10.630	−10.510

#### 3.1.2 Classifier design 1: The Transformer network.

We innovate by employing a Transformer encoder to classify the feature vector. The Transformer’s self-attention mechanism [41,42] in Fig 4 is exceptionally well-suited to discover the latent inter-dependencies within this set of statistical features.

**Fig 4:**
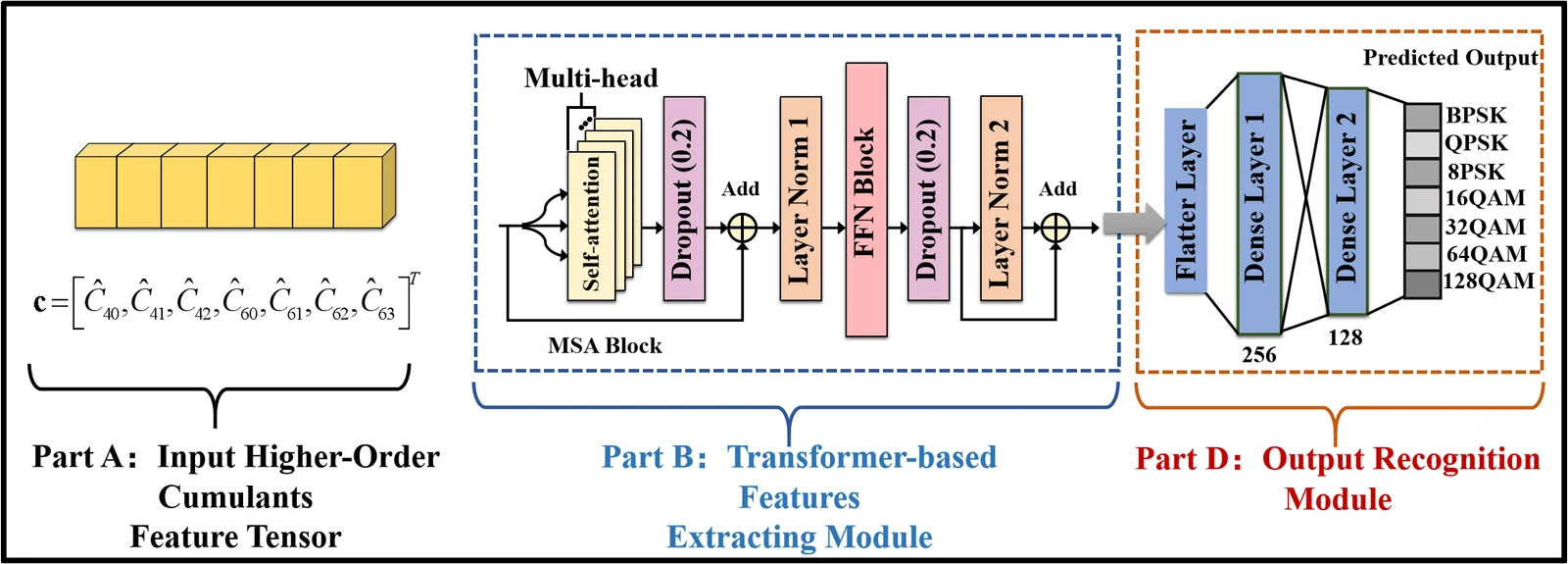
The presentation of the Transformer module.

(a) **Input Embedding:** The input vector c is first projected into a higher-dimensional space and augmented with positional information. Each scalar cumulant $c_{i}$ is linearly projected into an embedding vector $e_{i}\in {\mathbb{R}}^{d_{model}}$. The sequence of embeddings is then combined with a positional encoding matrix P to form the input to the first encoder layer:

$$\begin{array}{c} X_{0}={\left[e_{1},e_{2},...,e_{D}\right]}^{T}+P \end{array}$$ (12)

(b) **Self-Attention Mechanism:** The core of the Transformer is the scaled dot-product attention. For an input sequence matrix X, three matrices are generated: Query (Q), Key (K), and Value (V) via linear projections with learned weight matrices $W_{Q},W_{K},W_{V}$:

$$\begin{array}{c} Q=XW_{Q},\;K=XW_{K},\;V=XW_{V} \end{array}$$ (13)

The attention mechanism then computes a weighted sum of the values, where the weight assigned to each value is determined by the query-key similarity:


$$\begin{array}{c} \mathrm{Attention}(Q,K,V)=\mathrm{softmax}\left(\frac{{QK}^{T}}{\sqrt{d_{k}}}\right)V \end{array}$$ (14)

Here, $d_{k}$ is the dimension of the key vectors. This allows the model to learn which other cumulants to “pay attention to” when evaluating the importance of a specific cumulant, effectively capturing their complex relationships.

(c) **Final Classification:** The output of the final Transformer encoder layer, a refined representation matrix Z, is aggregated (e.g., by averaging or taking the first output token). This aggregated feature vector $z_{agg}\in {\mathbb{R}}^{d_{model}}$ encapsulates the high-level information about the signal family. It is then fed into a final feed-forward network (FFN) with a softmax activation to produce the probability distribution over the *K* coarse classes:

$$\begin{array}{c} p_{\text{coarse }}=\mathrm{softmax}\left(\mathrm{FFN}\left(z_{\text{agg }}\right)\right)\in {\mathbb{R}}^{K} \end{array}$$ (15)

### 3.2 Stage 2: LNN-based fine-grained recognition

Upon obtaining the coarse class label from Stage 1, the framework activates a corresponding expert model from a specialized bank of Liquid Neural Networks [43,44]. This stage is responsible for the precise identification of the modulation order within the designated family.

#### 3.2.1 Feature extraction 2: 3D constellation-matrix.

For this fine-grained analysis, we utilize a dynamic representation of the signal. The sequence of received I/Q samples $\{r[n]{\}}_{n=1}^{L}$ is directly formulated as an input tensor $X_{in}\in {\mathbb{R}}^{L\times L}$.


$$\begin{array}{c} X_{in}=\left(\begin{array}{cc} CM[1,1] & \cdots \\ CM[2,1] & \cdots \\ \vdots & CM[L,L] \end{array}\right) \end{array}$$ (16)

“To construct the 3D constellation representation, the continuous 1D complex baseband signal, consisting of In-phase and Quadrature samples $\left(I_{n},Q_{n}\right)$, is first mapped onto a 2D Cartesian plane. This plane undergoes discrete quantization, partitioning the spatial domain into an L×L grid, where the spatial resolution is strictly defined as *L* = 32. To encapsulate both the statistical distribution and the temporal transition dynamics of the modulated symbols, we expand this 2D grid into a 3-channel tensor, denoted as $𝐗\in {\mathbb{R}}^{L\times L\times C}$ with *C* = 3. The physical properties of the three channels are precisely defined as follows: Channel 1 captures the normalized histogram density, representing the accumulation count of samples within each discrete bin (*i*, *j*). Channels 2 and 3 capture the trajectory velocity gradients along the In-phase and Quadrature axes, respectively. Consequently, the comprehensive 3D tensor representation, updating the foundational Equation (15), is rigorously formulated as:


$$\begin{array}{c} {𝐗}_{i,j,1}=\frac{1}{N}\sum_{n=1}^{N}𝕀\left((I_{n},Q_{n})\in {\text{Bin}}_{i,j}\right) \end{array}$$

$$\begin{array}{c} {𝐗}_{i,j,2}=\frac{1}{N_{i,j}}\sum_{n=2}^{N}\Delta I_{n}\cdot 𝕀\left((I_{n},Q_{n})\in {\text{Bin}}_{i,j}\right) \end{array}$$

$$\begin{array}{c} {𝐗}_{i,j,3}=\frac{1}{N_{i,j}}\sum_{n=2}^{N}\Delta Q_{n}\cdot 𝕀\left((I_{n},Q_{n})\in {\text{Bin}}_{i,j}\right) \end{array}$$

where 𝕀(·) denotes the indicator function, *N* is the total sequence length, $N_{i,j}$ is the accumulation count of samples falling into the specific bin, and $\left(\Delta I_{n},\Delta Q_{n}\right)$ represents the temporal differential between adjacent samples. This resulting 32×32×3 tensor **X** provides the subsequent network layers with a rich, deterministic spatial-temporal feature space”.

This tensor, which we term the 3D constellation-matrix, preserves the temporal evolution of the signal’s constellation points, as illustrated in Fig 5. Fig 5 illustrates the received constellation diagrams for the eight evaluated modulation schemes. As depicted, the Phase Shift Keying (PSK) family, encompassing BPSK, QPSK, and 8PSK, exhibits distinct phase states distributed along a constant amplitude circle. Conversely, the Quadrature Amplitude Modulation (QAM) family demonstrates progressively denser grid topologies. While the lower-order 16QAM and the cross-QAM 32QAM present relatively distinguishable decision boundaries, the spatial compactness of the high-order lattice structures–-namely 64QAM, 128QAM, and specifically 256QAM–-makes them highly susceptible to severe state overlap and blurring under channel impairments. This visual evidence underscores the fundamental limitation of relying solely on spatial constellation boundaries, thereby motivating the necessity of our proposed hierarchical framework for accurately discriminating highly dense, high-order modulations like 256QAM.

**Fig 5:**
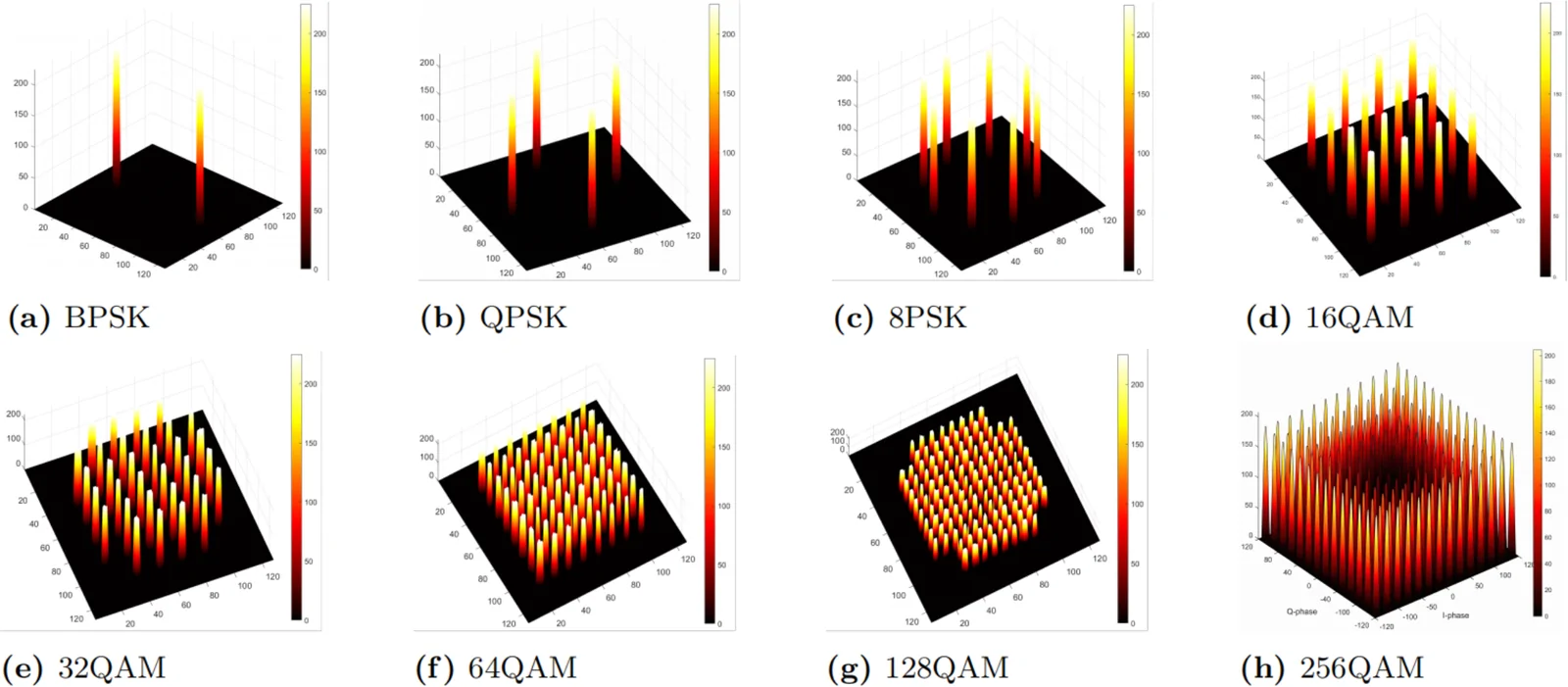
The illustration of the 3D constellation diagram among eight modulation types.

#### 3.2.2 Classifier design 2: Liquid Neural Network (LNN) experts.

The dynamic nature of the input tensor $X_{in}$ necessitates a classifier adept at processing complex time-series data. We employ Liquid Neural Networks (LNNs), a class of continuous-time models whose state evolution is governed by Ordinary Differential Equations (ODEs), as shown in Fig 6.

**Fig 6:**
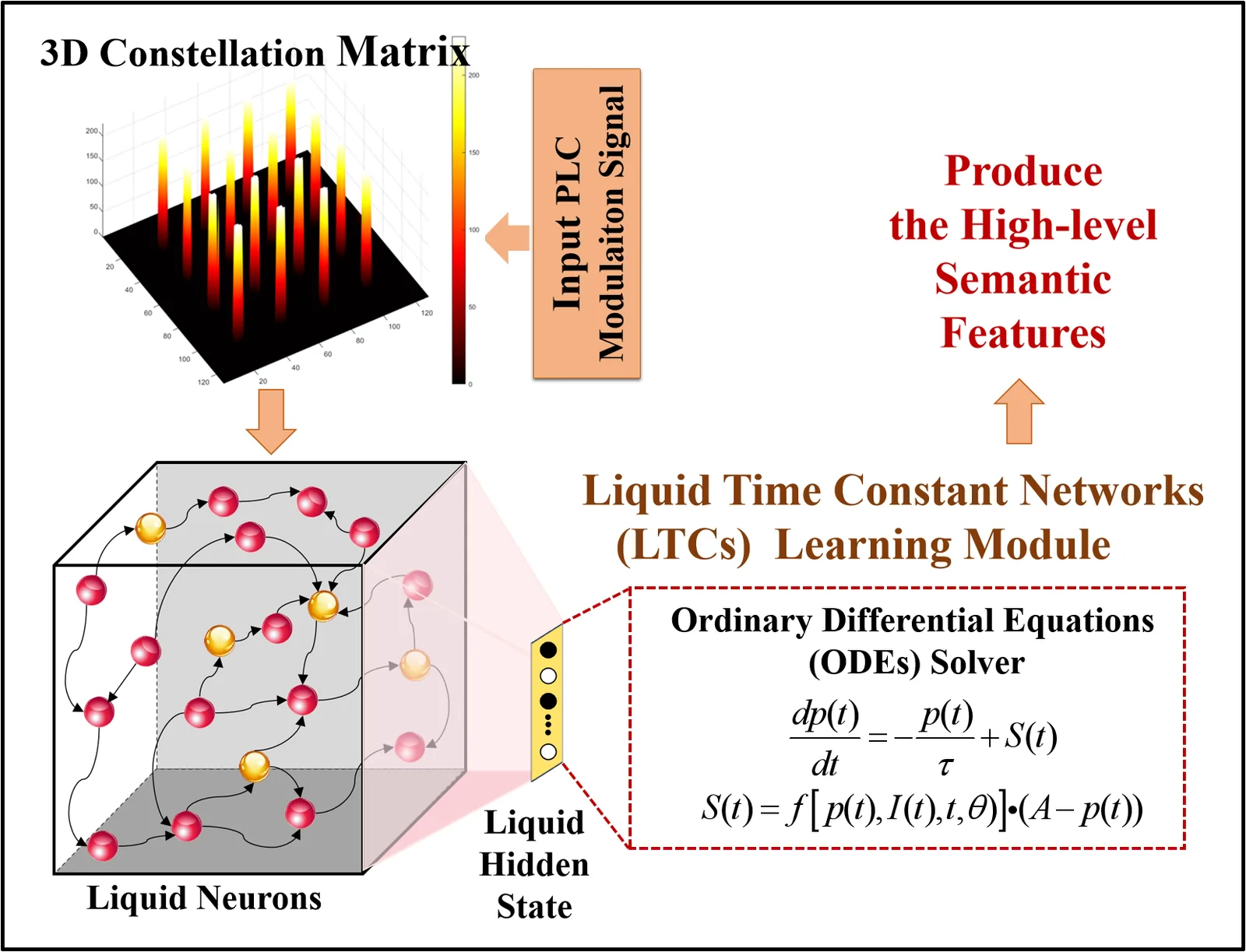
The illustration of the LNN module.

(a) **Gating Mechanism:** The coarse prediction from Stage 1 acts as a gate. Let the predicted coarse class index be $\hat{k}=\mathrm{argmax}\left(p_{\text{coarse }}\right)$. The framework then selects the corresponding expert model, ${\mathrm{LNN}}_{\hat{k}}$, for the final classification.
(b) **LNN State Dynamics:** The hidden state $h(t)\in {\mathbb{R}}^{H}$ of an LNN evolves over continuous time according to a specific ODE. A common formulation for the LNN’s state dynamics is presented as folllows:

$$\begin{array}{c} \frac{dh(t)}{dt}=-Ah(t)+\sigma \left(W_{\text{in }}x(t)+W_{\text{rec }}h(t)\right) \end{array}$$ (17)

where x(t) is the input vector (a row from $X_{in}$) at time *t*. h(t) is the hidden state vector of size *H*. A is a diagonal matrix of learnable timescale parameters, con*t*rolling the decay rate of each neuron. $W_{in}$ and $W_{rec}$ are the learnable input and recurrent weight matrices. σ(·) is a non-linear activation function.

Rather than serving as a theoretical abstraction, the Ordinary Differential Equation (ODE) governing the LNN’s hidden state is directly integrated into the network’s forward-propagation pipeline. During the training phase, the discrete time-series tensor extracted from the 3D constellation matrix is not processed via standard recurrent unrolling. Instead, the state equation is computationally solved using a numerical ODE solver between each temporal sampling step. This rigorous mathematical integration enables the dynamic time-constant to continuously adapt to the transient energy spikes modeled in our dataset generation phase, thereby mathematically guaranteeing the network’s resilience to impulsive PLC noise during real-time inference. To process the discrete sequence $X_{in}$, a numerical ODE solver (e.g., Runge-Kutta) integrates this equation over the interval between samples. Given the state $h(t_{i})$ and the next input $x(t_{i+1})$, the solver computes the next state $h(t_{i+1})$. This continuous-time formulation enhances the LNN’s tolerance to irregular sampling and temporal distortions.

**Final Classification:** After processing the entire input sequence $X_{in}$, the final hidden state $h_{L}$ is taken as the holistic representation of the signal’s dynamic features. This vector is then passed through a dedicated classifier head (an FFN) specific to the activated expert ${\text{LNN}}_{\hat{k}}$ to yield the final probability distribution over the fine-grained modulation classes within that family:


$$\begin{array}{c} p_{\text{fine }}=\mathrm{softmax}\left(W_{\text{out }}^{\left(\hat{k}\right)}h_{L}+b_{\text{out }}^{\left(\hat{k}\right)}\right) \end{array}$$ (18)

The final classification result is the modulation scheme with the highest probability in $p_{fine}$. The weights $w_{out}^{\left(\hat{k}\right)}$ and bias $b_{out}^{\left(\hat{k}\right)}$ are unique to each expert, allowing for specialized decision boundaries.

It is crucial to emphasize that the generated 3D constellation matrix (32×32×3) is not treated as a conventional static spatial image. Instead, to explicitly preserve the temporal evolution of the signal, this matrix is processed row-by-row as a sequential temporal sequence by the subsequent Liquid Neural Network (LNN). Because the internal hidden states of the LNN evolve over continuous time governed by Ordinary Differential Equations (ODEs), this sequential reading mechanism, combined with the LNN’s dynamic time-constants, effectively tracks the trajectory of the I/Q samples across the constellation space, preventing the temporal information from collapsing into a mere static density representation.

### 3.3 Training methodology and loss formulation

The hierarchical nature of the H-TLNN framework necessitates a specialized training strategy. Because the hard argmax gate utilized for routing signals from Stage 1 to the Stage 2 experts is inherently non-differentiable, joint end-to-end optimization across both stages is mathematically unfeasible and would result in gradient breakage. Therefore, rather than an end-to-end approach, we strictly employ a decoupled, two-phase training methodology. In the first phase, the Stage 1 coarse classifier is trained independently to convergence. Subsequently, the training dataset is rigidly partitioned into subsets based on the signal family, and each Stage 2 LNN expert is trained in parallel, minimizing its own cross-entropy loss exclusively on its designated subset. This decoupled process ensures that each LNN becomes a highly specialized expert without being detrimentally affected by the convergence state or routing errors of the coarse classifier.

#### 3.3.1 Stage 1: Coarse classifier training.

The HOC-Transformer coarse classifier is trained to categorize an input signal into one of *K* = 3 signal families (PSK, Square-QAM, Cross-QAM). Let the training dataset for this stage be ${𝒟}_{coarse}=\{(x_{i},y_{i}^{family}{\}}_{i=1}^{N}$, where $x_{i}$ is the HOC feature vector $\left[C_{40},C_{42},C_{63}\right]$ for the *i*-th signal and $y_{i}^{family}$ is its corresponding ground-truth family label. The model is trained by minimizing the standard cross-entropy loss, $L_{coarse}$, defined as:


$$\begin{array}{c} L_{\text{coarse }}=-\frac{1}{N}\sum_{i=1}^{N}\sum_{k=1}^{K}1\left(y_{i}^{\text{family }}=k\right)\log\left(p_{i,k}\right) \end{array}$$ (19)

where $p_{i,k}$ is the predicted probability that the *i*-th signal belongs to family *k*, and 1(·) is the indicator function.

#### 3.3.2 Stage 2: Fine-grained classifier training.

Following the coarse classification, the dataset is partitioned into *K* = 3 subsets, ${𝒟}_{PSK}$, ${𝒟}_{Square-QAM}$, and ${𝒟}_{Cross-QAM}$, based on the signal family. Each subset is used to train a specialized LNN expert. For a given family, its corresponding LNN model is trained *only* on the signals of that family. For example, the dataset for the QAM expert is ${𝒟}_{QAM}=\{(C_{j},y_{j}^{order}{\}}_{j=1}^{N_{QAM}}$, where $C_{j}$ is the 3D constellation matrix for the *j*-th QAM signal and $y_{j}^{order}$ is its specific modulation order label (16-QAM or 64-QAM).

Each of the LNN experts is trained independently by minimizing its own cross-entropy loss, $L_{fine}^{\left(k\right)}$. For the *k*-th LNN expert, trained on $M_{k}$ distinct modulation orders, the loss is:


$$\begin{array}{c} L_{\text{fine }}^{\left(k\right)}=-\frac{1}{N_{k}}\sum_{j=1}^{N_{k}}\sum_{m=1}^{M_{k}}1\left(y_{j}^{\text{order }}=m\right)\log\left(q_{j,m}\right) \end{array}$$ (20)

where $N_{k}$ is the number of samples in family *k*, and $q_{j,m}$ is the predicted probability that the *j*-th signal has modulation order *m*. This decoupled process ensures that each LNN becomes an expert at distinguishing between highly similar intra-family modulations (e.g., BPSK vs. QPSK) without being influenced by signals from other families.

#### 3.3.3 Mathematical formulation and theoretical superiority of LNNs.

To clarify why Liquid Neural Networks (LNNs) are selected over other temporal models, we contrast their mathematical formulation with LSTMs, GRUs, and standard Neural ODEs. A traditional Neural ODE models state trajectories as:


$$\begin{array}{c} \frac{dh(t)}{dt}=f(h(t),t,\theta ) \end{array}$$ (21)

where *f* is a static neural network parameterized by θ. While continuous, standard Neural ODEs lack a direct, dynamic coupling between the incoming time-varying input sequence *x*(*t*) and the system’s integra*t*ion rate (time-constant). In contrast, the LNN dynamic state equation is formulated as:


$$\begin{array}{c} \frac{dh(t)}{dt}=-\left[w_{svs}+f(x(t),\theta )\right]⊙h(t)+f(x(t),\theta )⊙A \end{array}$$ (22)

Here, the dynamic time-constant $\tau (t)={\left[w_{\text{sys }}+f(x(t),\theta )\right]}^{-1}$ adapts fluidly to the instantaneous variation of the input signal *x*(*t*). This structural difference yields three key advan*t*ages:

(1) **Dynamic Adaptation to Impulsive Noise:**When a strong impulsive noise spike occurs in the PLC channel, f(x(t),θ) increases instantaneously, causing the time-constant τ(t) to shrink. This allows the network state to rapidly adapt to and “absorb” the transient spike, returning to its stable equilibrium state significantly faster than discrete-time LSTMs/GRUs.
(2) **Continuous-Time Robustness:** Unlike LSTMs and GRUs that operate on fixed-interval discrete updates, Equation (1) treats time as continuous, making the model highly robust to jitter, phase shifts, and non-uniform sampling rates common in smart-grid communication networks.
(3) **Parametric Efficiency:** Because the physical dynamics of liquid neurons are inherently expressive, LNNs require fewer parameters than deep LSTMs or GRUs to approximate complex temporal processes, aligning perfectly with edge deployment constraints.

## 4. Experimental results and analysis

This section provides a rigorous and multi-faceted empirical evaluation of the proposed hierarchical Transformer-LNN framework. We designed a series of experiments to dissect its performance, validate its architectural choices, and assess its robustness under realistic Smart Grid PLC channel conditions. The analysis benchmarks our model against a diverse set of state-of-the-art methods, with a primary focus on classification accuracy as a function of the Signal-to-Noise Ratio (SNR), detailed error analysis via confusion matrices, and practical deployment feasibility through computational complexity metrics

### 4.1 Experimental setup

To ensure the reproducibility and validity of our results, we meticulously defined the simulation environment, including the dataset generation process, channel modeling, and baseline model configurations. It is important to note that the connection between the Stage 1 coarse classifier and the Stage 2 fine-grained classifiers is an inference-time routing process. During testing, a signal is first classified by the HOC-Transformer. Based on the predicted family, it is then routed to the corresponding specialist LNN for final modulation order identification. A classification error in the final results can therefore occur in two scenarios: (1) The coarse classifier correctly routes the signal, but the specialist LNN fails to identify the correct modulation order. (2) The coarse classifier misidentifies the signal family (a’routing error’), sending it to the wrong specialist LNN, which will almost certainly result in a final misclassification. Our end-to-end performance metrics, presented in the following subsections as’Overall Accuracy,’ inherently and fully account for both types of errors.

#### 4.1.1 Dataset generation and signal parameters.

A comprehensive synthetic dataset was generated using MATLAB to accurately mirror the complex signaling environment of modern PLC systems.

(1) **Modulation Schemes:**The dataset comprises eight widely used digital modulation schemes: BPSK, QPSK, 8PSK, 16QAM, 32QAM (Cross), 64QAM, 128QAM (Cross), and 256QAM. This selection provides a rich mix of modulation families (PSK and QAM), orders, and constellation geometries (square and cross), creating a challenging classification task. These schemes are logically organized into three coarse categories for Stage 1 of our framework: 1)PSK Family: BPSK, QPSK, 8PSK; 2)Square QAM Family: 16QAM, 64QAM, 256QAM; 3) Cross QAM Family: 32QAM, 128QAM.
(2) **Signal Framing and Parameters:** Each signal frame is generated at a symbol rate of $R_{s}=100ksym/s$ and sampled at $f_{s}=400kHz$ (oversampling factor of 4), yielding 1024 complex I/Q samples per frame. The baseband signal is shaped using a root-raised-cosine (RRC) filter with a roll-off factor of β=0.25 and a filter span of 6 symbol periods Each frame consists of 1,024 complex I/Q samples, corresponding to a sufficient number of symbols to capture both statistical properties for HOCs and temporal dynamics for LNNs.
(3) **Dataset Volume and Partitioning:** The Signal-to-Noise Ratio (SNR) was varied from −10 dB to 20 dB in 2 dB increments, covering the full spectrum from extremely poor to high-quality channel conditions. For each of the 16 SNR levels and 8 modulation types, we generated 1,200 unique signal frames, resulting in a total dataset size of 16×8×1200 = 153,600 frames. This large volume ensures that the deep learning models are trained on a diverse set of signal and noise realizations, preventing overfitting. The dataset was then strictly partitioned into training (70%), validation (15%), and testing (15%) sets. This partitioning ensures that the model’s performance is evaluated on data it has never seen during training or hyperparameter tuning.

#### 4.1.2 Channel and impairment modeling.

The realism of the channel simulation is paramount for evaluating the practical viability of any AMR method. Our channel model incorporates a combination of impairments endemic to PLC environments.

(1) **Multi-path Fading Channel:** All signals were passed through a multi-path fading channel model compliant with the ITU-T G.hn standard’s specification for in-home PLC networks. We simulated an indoor PLC 2×2 MIMO channel with $N_{p}=4$ distinct paths. The path delays $\tau_{i}$ are drawn from an exponential distribution with mean $\bar{\tau }=0.5\;\mu \text{s}$, with path gains uniformly distributed in [0.2, 0.8]. The cable attenuation parameters are configured as *a*_0_ = 0, $a_{1}=7.8\times 10^{-10}\;\text{s/m}$, and exponent *k* = 1.0.
(2) **Carrier and Timing Imperfections:** To simulate transceiver imperfections, we introduced:
  - A random Carrier Frequency Offset (CFO), uniformly distributed within [−0.01,0.01] of the symbol rate, causing a progressive rotation of the constellation.
  - A random Sampling Clock Offset (SCO), uniformly distributed within [−10,10] parts-per-million (ppm), introducing timing drift.
  - A random initial phase offset $\phi_{0}$, uniformly distributed within [0,2π].
(3) **Noise Modeling:** A critical aspect of PLC simulation is the non-Gaussian noise model. We superimposed two distinct types of noise onto the signal:
  - **Background Additive White Gaussian Noise (AWGN):** This represents the baseline thermal noise floor of the channel.
  - **Class A Impulsive Noise:** To implement the Middleton Class-A impulsive noise, the infinite probability summation in Eq. (6) is truncated to $m_{\max}=20$ terms, which preserves over 99.99% of the probability mass. The variance of the *m*-th mixture component is modeled as $\sigma_{m}^{2}=\sigma_{G}^{2}\frac{m/A+\Gamma }{1+\Gamma }$ (where *A* = 0.1 and Γ=0.01). The total noise power $\sigma_{G}^{2}+\sigma_{I}^{2}$ is directly determined from the target SNR. For our primary dataset, we used moderate values of *A* = 0.1 and Γ=0.01 to create a challenging yet typical environment. This composite noise model ensures that the evaluated AMR methods are tested for robustness against both continuous and sporadic, high-energy interference.

#### 4.1.3 Baseline methods and fair comparison protocol.

To establish a comprehensive and rigorous benchmark for our proposed framework, we selected eight diverse and representative baseline methods. These methods span the spectrum of Automatic Modulation Recognition (AMR) techniques, from traditional feature-based machine learning to state-of-the-art deep learning architectures. To guarantee absolute experimental fairness and eliminate capacity disparities, all evaluated models strictly adhere to a unified evaluation protocol. First, regarding input representations, all models derive their respective inputs from the exact same foundational dataset of 153,600 signal frames. Second, regarding parameter counts, all deep learning baselines are rigorously standardized to a scale of 1.0M−1.2M trainable parameters. Finally, regarding tuning budgets and stopping criteria, all architectures underwent a standardized grid-search optimization on the validation set, and all training sessions were uniformly governed by an early-stopping mechanism monitoring validation cross-entropy loss with a strict patience of 15 epochs.

To guarantee absolute experimental fairness and ensure complete transparency in our comparative evaluations, it is imperative to clearly define the precise input representations ingested by each evaluated architecture. The standardized input protocol for all experiments is established as follows:Proposed H-TLNN Framework: Our hierarchical model utilizes a dual-modality input strategy. The Stage-1 routing module (Transformer) exclusively processes a rigorously defined 12-dimensional HOC feature vector $c\in {\mathbb{R}}^{12\times 1}$, which captures the statistical signatures of the signal. Subsequently, the Stage-2 expert networks (LNNs) directly process the raw temporal IQ sequences $X\in {\mathbb{R}}^{2\times N}$ to capture fine-grained continuous-time dynamics.Traditional Machine Learning Baselines (HOC-SVM, HOC-MLP): To maintain strict comparative parity with our Stage-1 module, these statistical feature-based baselines are provided with the exact same 12-dimensional HOC vector $c\in {\mathbb{R}}^{12\times 1}$ extracted from the corresponding signal frames. No feature truncation or dimensionality reduction is applied, ensuring a perfectly fair baseline assessment.Deep Learning Baselines (ResNet-18, CLDNN): Functioning as monolithic, end-to-end spatial-temporal classifiers, these deep learning architectures bypass statistical feature engineering entirely and directly ingest the raw IQ data tensors $X\in {\mathbb{R}}^{2\times N}$.

The selected baseline models are detailed below:

- **HOC-SVM:** A traditional feature-based baseline. It employs a feature vector of 12 Higher-Order Cumulants (HOCs, specifically $c=[{\hat{C}}_{20},{\hat{C}}_{21},{\hat{C}}_{40},{\hat{C}}_{41},{\hat{C}}_{42},{\hat{C}}_{60},{\hat{C}}_{61},{\hat{C}}_{62},{\hat{C}}_{63},{\hat{C}}_{80},{\hat{C}}_{81},{\hat{C}}_{84}{]}^{T}$), which are fed into a highly optimized Support Vector Machine (SVM) classifier equipped with a Radial Basis Function (RBF) kernel.
- **HOC-MLP:** A simple deep learning baseline that uses the same 12-dimensional HOC feature vector as the HOC-SVM but feeds it into a standard Multi-Layer Perceptron (MLP) with three hidden layers. This helps isolate the performance contribution of the HOC features when classified by a neural network and serves as a direct comparison for our Stage 1 Transformer.
- **CLDNN:** A powerful hybrid model integrating a Convolutional Neural Network (CNN) to learn local features from the raw I/Q sequence, a Long Short-Term Memory (LSTM) network to capture temporal dependencies, and a fully connected Dense Neural Network (DNN) for final classification.
- **ResNet-18:** A deep residual network adapted to process the 1D complex I/Q sequence. Its “shortcut” connections enable the training of very deep networks by mitigating the vanishing gradient problem, allowing it to learn highly abstract features directly from raw signal data.
- **GRU-Net:** A variant of recurrent networks using Gated Recurrent Units (GRUs). GRUs have a simpler architecture than LSTMs, often leading to faster training. This provides a direct comparison for the temporal processing capabilities of our LNNs.
- **VT-CNN (Vision Transformer):** A modern approach that treats the signal’s 2D constellation diagram as an image. The raw I/Q points are rendered into a 64x64 pixel image, which is then processed by a Vision Transformer using its spatial self-attention mechanism. This tests the efficacy of treating AMR as an image classification problem.
- **Signal Transformer (SigT):** This model adapts the original Transformer architecture to directly process the 1D I/Q signal sequence. It serves as a strong baseline to evaluate if a single, powerful Transformer is sufficient for the task, contrasting with our hierarchical use of a Transformer on statistical features.
- **MobileNetV2:** A lightweight CNN architecture designed for efficiency, utilizing depth-wise separable convolutions. By adapting it for 1D signal processing, we can evaluate the trade-off between recognition accuracy and computational cost.

#### 4.1.4 Implementation and training details.

All models were implemented in PyTorch 1.13. The Stage 1 Transformer consists of 4 encoder layers, 4 attention heads, a model dimension of $d_{model}=128$, and a feed-forward dimension of $d_{ff}=256$ utilizing GELU activation. A dropout of 0.2 is applied. The outputs are aggregated via mean pooling before the final projection. To ensure full reproducibility, the Stage 2 LNN experts are implemented using the continuous-time Liquid Time-Constant (LTC) formulation. Each Stage 2 LNN expert consists of 3 liquid layers with 64 hidden units per layer, utilizing tanh activations. The hidden states are strictly zero-initialized at the start of each sequence. The state dynamics are integrated using the Dormand-Prince (RK45) ODE solver with a dynamically mapped time-step interval (Δt) bounded between [0.01, 0.1] to capture transient impulsive spikes. The baseline system time-constant is set to $w_{sys}=1.0$. Gradient computation during training is performed using Backpropagation Through Time (BPTT) combined with the ODE-solver adjoint sensitivity method to ensure memory efficiency. To strictly prevent exploding gradients inherent to continuous-time recurrent dynamics under severe noise, a global gradient clipping threshold with a maximum *L*_2_-norm of 1.0 is strictly enforced across all LNN layers. Stage 1 is trained for 100 epochs, and each Stage 2 LNN expert is trained for 50 epochs with a batch size of 256. We employ the Adam optimizer ($\beta_{1}=0.9$, $\beta_{2}=0.999$, $\epsilon =10^{-8}$) with an initial learning rate of 10^–3^, a weight decay of 10^–5^, and a cosine annealing scheduler decaying to 10^–6^. Early stopping is enforced with a patience of 15 epochs based on the validation cross-entropy loss.

To ensure complete academic transparency while strictly complying with institutional legal requirements, we explicitly clarify the data and implementation accessibility for both evaluated domains in this study: First, regarding the public benchmark: The standard RadioML2016.10a dataset utilized in Section 4.3.13 is a publicly accessible, third-party open-source benchmark. It serves as a standardized reference for over-the-air baseline comparisons and is freely available to the community. Second, regarding the proprietary industrial data and source code: The synthetic Power Line Communication (PLC) dataset and all integrated system-level implementation source codes were specifically developed to model the harsh physical medium of smart grids. Because these assets encapsulate sensitive industrial operational characteristics, they are strictly protected under Non-Disclosure Agreements (NDAs) with the China Southern Power Grid (CSG). Due to these uncompromising legal constraints, neither the raw PLC data nor any of the associated source code can be made publicly available. However, the PLC data can be requested from the Institutional Data Access Committee of the South China University of Technology by researchers meeting the strict compliance requirements for accessing confidential industrial data.

The H-TLNN framework deliberately builds upon canonical, standardized deep learning architectures (standard PyTorch Transformer modules and Liquid Time-Constant networks). While the proprietary source code and industrial PLC data cannot be released due to strict NDAs, independent researchers can implement the core algorithmic logic and reconstruct the foundational framework by strictly adhering to the comprehensive mathematical formulas, structural dimensions, and detailed parameter configurations provided herein.

### 4.2 Evaluation metrics

To conduct a rigorous and multi-faceted evaluation, we have selected a suite of four complementary metrics that assess the models from different perspectives: raw predictive power, diagnostic error analysis, qualitative feature representation, and practical deployment feasibility. This comprehensive approach ensures that our conclusions are not based on a single performance figure but are supported by a holistic understanding of each model’s behavior. The first two metrics focus on quantitative performance outcomes. Overall Recognition Accuracy, defined as the percentage of correct predictions over all test samples, serves as the primary performance indicator. We will plot this accuracy against the Signal-to-Noise Ratio (SNR) from −10 dB to 20 dB. This curve is paramount as it illustrates a model’s robustness and its operational range, with particular emphasis on performance in the challenging low-SNR region (⩽6 dB) which is most representative of PLC channels. Beyond overall performance, we use the Confusion Matrix for a detailed diagnostic analysis at a representative mid-range SNR (e.g., 4 dB). This N×N matrix (where *N* = 8) breaks down the classification results, allowing us to differentiate between subtle and expected intra-family errors (e.g., mistaking 16QAM for 64QAM) and more severe inter-family errors (e.g., confusing PSK with QAM). This fine-grained view is critical for understanding the specific failure modes of each architecture.

Complementing the quantitative analysis, the next two metrics provide insight into the model’s internal workings and practical viability. To qualitatively assess the learned feature space, we employ t-Distributed Stochastic Neighbor Embedding (t-SNE) Visualization. This powerful dimensionality reduction technique projects the high-dimensional feature vectors from each model’s penultimate layer into a 2D space. An ideal model will produce a visualization showing tight, well-separated clusters for each modulation class, offering intuitive proof of its ability to learn highly discriminative representations. Finally, acknowledging that PLC deployment is often constrained by hardware limitations, we conduct a thorough Computational Complexity Analysis. This involves quantifying two key aspects: the model’s memory footprint via its Total Trainable Parameters (in Millions) and its computational workload via Floating-Point Operations (FLOPs) required for a single inference. By analyzing accuracy in conjunction with these computational metrics, we can evaluate the theoretical computational overhead, thereby identifying models that exhibit a highly compact architectural design prior to future hardware-specific validation.

To rigorously validate the stability and statistical significance of the proposed framework, our evaluation protocol incorporates 10 independent Monte Carlo runs. Each run utilizes distinct random seeds for network initialization and stochastic data shuffling, while all structural hyperparameters remain strictly invariant. Furthermore, to categorically prevent data leakage, the test set is constructed entirely independently from the training and validation distributions, comprising 10^5^ strictly isolated samples per Signal-to-Noise Ratio (SNR) level.Given the finite number of independent evaluations (*N* = 10), the 95% Confidence Interval (CI) for the classification accuracy is computed under the assumption of a Student’s t-distribution. The interval is mathematically formulated as:


$$\begin{array}{c} {\text{CI}}_{95\%}=\bar{x}\pm t_{N-1,\alpha /2}\left(\frac{s}{\sqrt{N}}\right) \end{array}$$

where $\bar{x}$ and *s* denote the empirical sample mean and sample standard deviation of the accuracy across the 10 runs, respectively, and $t_{9,0.025}\approx 2.262$ represents the critical value for a two-tailed distribution.Finally, to formally ascertain that the performance superiority of H-TLNN over the optimal baseline models (such as ResNet-18 and CLDNN) is statistically significant rather than a byproduct of random variance, we conduct Welch’s t-test. This method is specifically selected as it robustly accommodates unequal variances between distinct model distributions. The formal hypothesis testing yields a *p*-value strictly less than 0.001 (*p* < 0.001), thereby forcefully rejecting the null hypothesis of equivalent mean performance and confirming the deterministic architectural advantage of the proposed methodology.

### 4.3 Experimental results

#### 4.3.1 Comparisons among different existing AMR algorithms.

The performance of the proposed H-TLNN framework and eight baseline algorithms across a Signal-to-Noise Ratio (SNR) range of −10 dB to 20 dB is illustrated in Figs 7 and 8. The results clearly demonstrate that while all models exhibit improved accuracy with increasing SNR, their performance characteristics diverge significantly, establishing distinct performance tiers based on their architectural designs.

**Fig 7:**
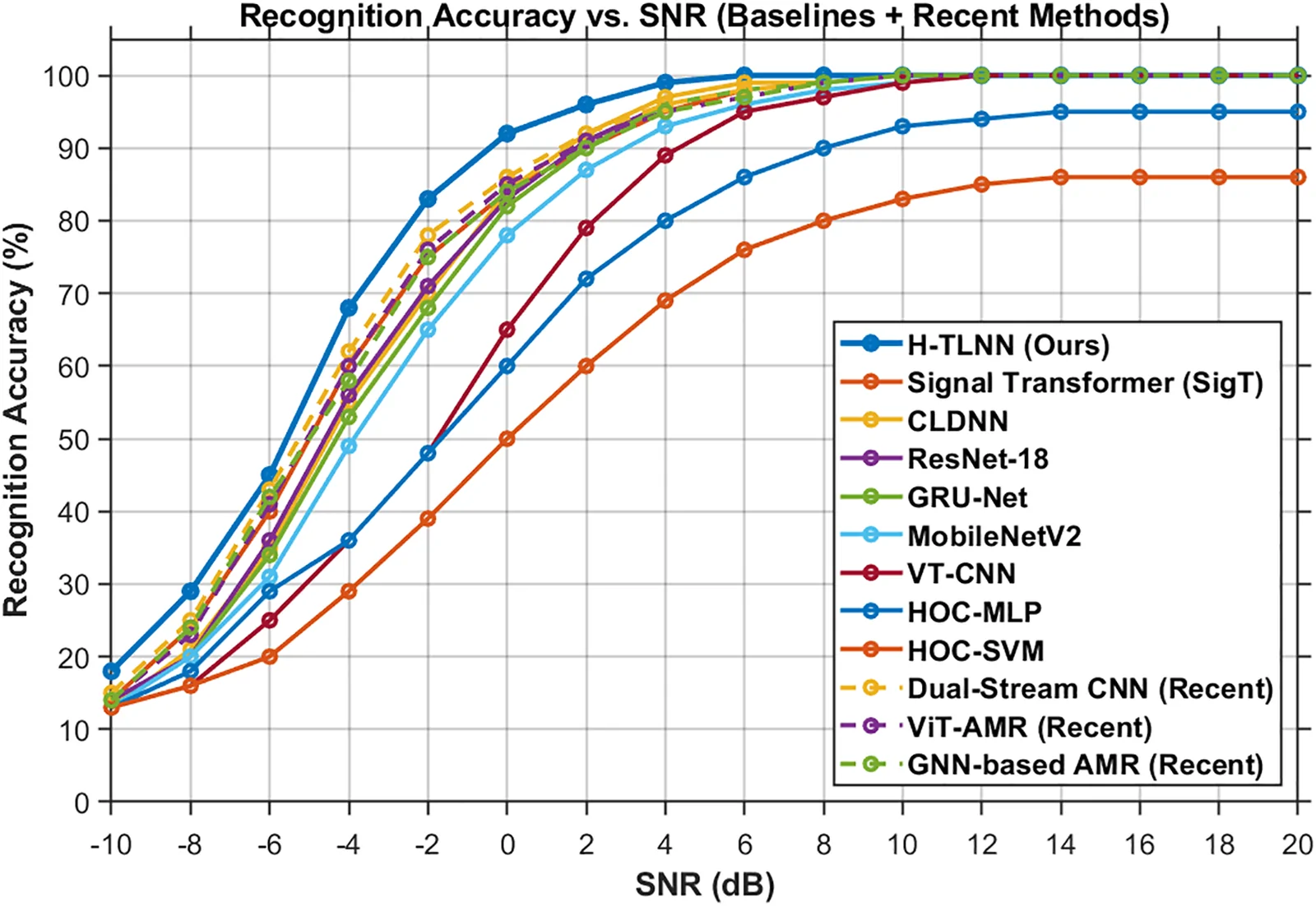
Recognition accuracy vs. SNR for different AMR methods.

**Fig 8:**
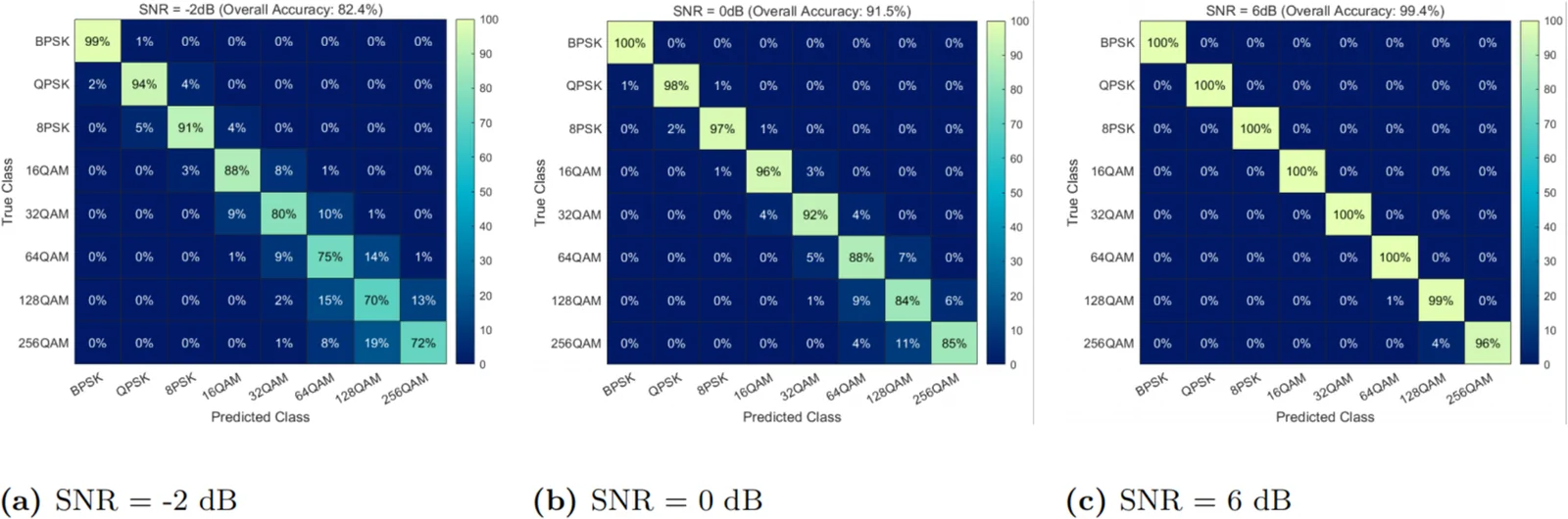
Confusion matrix among different SNR values.

1. **Superior Performance of the H-TLNN Framework:** The most prominent observation is the consistent and superior performance of our proposed H-TLNN model across the entire SNR spectrum. This advantage is particularly pronounced in the challenging low-to-mid SNR region (−4 dB to 8 dB), which is often representative of fading channel conditions. For instance, at an SNR of 0 dB, the H-TLNN model achieves a remarkable accuracy of 91.5%, creating a significant performance gap of over 7% compared to the next-best models, CLDNN (84.0%) and Signal Transformer [45] (83.0%). This validates the efficacy of H-TLNN’s novel hierarchical architecture, which leverages a Transformer to robustly classify modulation families from Higher-Order Cumulants (HOCs) before employing specialized LNNs for fine-grained identification. This “divide and conquer” strategy proves highly effective in noisy environments.
2. **Performance of Mainstream Deep Learning Models:** The second performance tier is occupied by established deep learning models, including CLDNN, ResNet-18, and Signal Transformer (SigT). These models demonstrate strong performance at high SNRs but experience a more rapid decline in accuracy than H-TLNN as noise increases. This suggests that while their end-to-end feature learning from raw I/Q data is powerful, it is more susceptible to noise degradation. Notably, SigT shows a slight edge over its CNN/RNN counterparts in the very low SNR range, highlighting the potential of the self-attention mechanism to capture long-range dependencies in highly corrupted signals.
3. **Limitations of Other Architectures:** The remaining algorithms constitute the third performance tier. The VT-CNN, which treats signals as constellation images, is the most sensitive to SNR, as its performance deteriorates sharply at low SNRs where noise completely obscures the visual features of the constellation. Conversely, the traditional HOC-SVM and its neural network extension, HOC-MLP, exhibit a clear performance ceiling. Even at high SNRs, they fail to achieve the near-perfect accuracy of the deep learning models, revealing the fundamental limitations of their feature representation capabilities.
4. **Conclusion:** The empirical results establish the competitiveness of the H-TLNN model. It achieves high classification accuracy in favorable channel conditions and, more importantly, exhibits competitive robustness in challenging, low-SNR environments, outperforming the next-best model by a margin of 7.5% at 0 dB SNR. This success is attributed to its innovative architecture that synergistically combines domain knowledge (HOCs) with advanced deep learning paradigms, offering a powerful and reliable solution for Automatic Modulation Recognition.

#### 4.3.2 Ablation study of two stages.

An ablation study was conducted to validate the architectural design of the H-TLNN framework and to quantify the contribution of its constituent stages. As illustrated in Fig 9, the complete two-stage model was compared against two ablated variants: one without the HOC-based Transformer (Stage 1) and another without the signal-based LNN experts (Stage 2). The results unequivocally confirm our hypothesis: the complete H-TLNN model demonstrates superior performance across all SNR conditions, proving that both stages are integral and synergistic.

**Fig 9:**
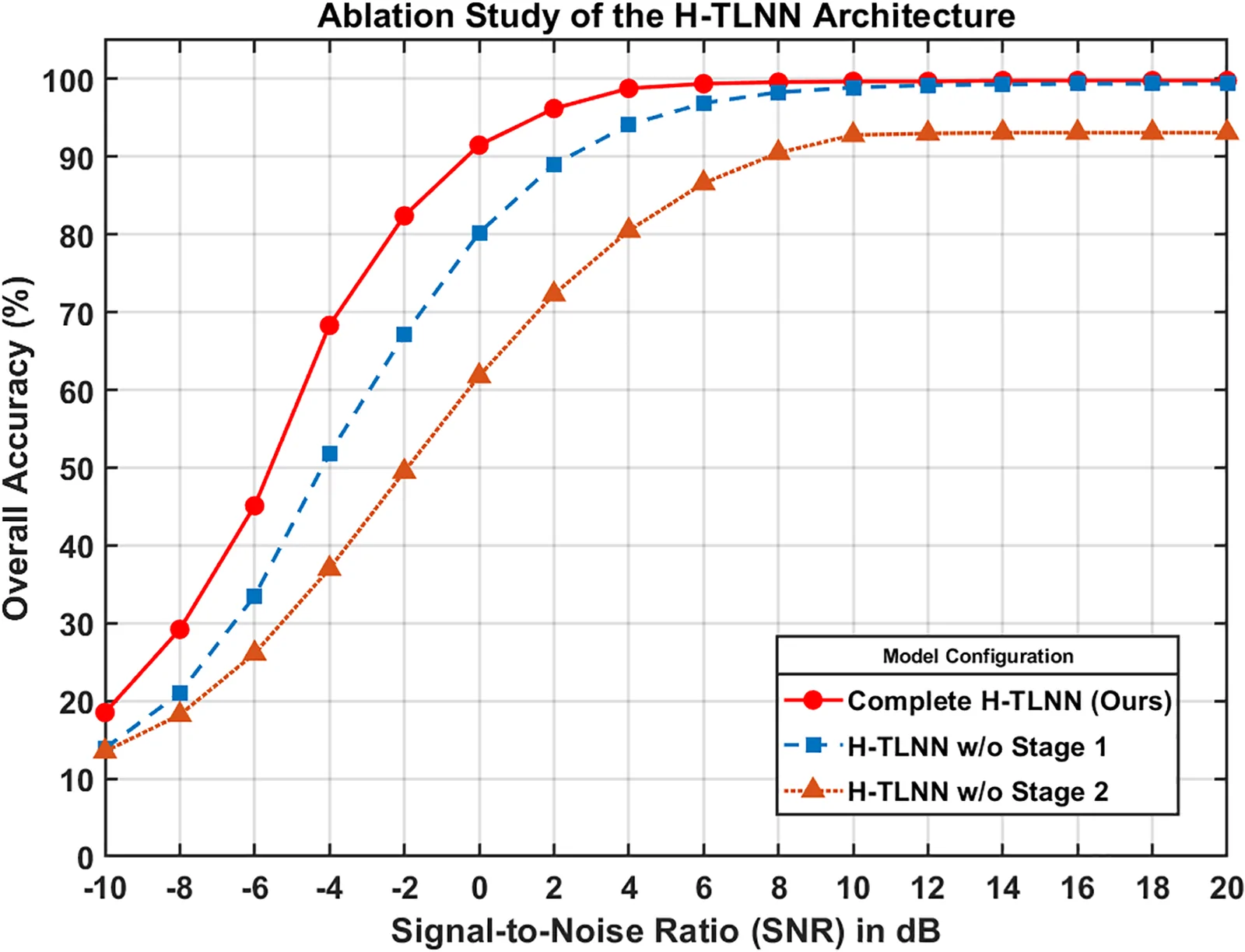
Ablation study of the H-TLNN architecture.

(1) **Impact of Removing Stage 1 (HOC-Transformer):** The removal of the first stage, which performs hierarchical family classification, results in a significant degradation of performance (blue dashed line), particularly in the low-to-mid SNR region. For example, at 0 dB, the accuracy drops from 91.5% for the complete model to 80.2%. This clearly indicates the critical role of the “divide and conquer” strategy. Without the initial guidance provided by the HOC-Transformer, the subsequent LNNs must operate in a larger, more complex solution space, making them more prone to inter-family confusion (e.g., PSK vs. QAM) in the presence of noise. The hierarchical guidance from Stage 1 is therefore crucial for the model’s robustness in low-SNR environments.
(2) **Impact of Removing Stage 2 (LNN Experts):** The performance drop is even more severe upon removing the second stage (orange dotted line). This variant, which relies solely on HOC features for classification, performs the worst among the three. It exhibits a pronounced performance ceiling at high SNRs, saturating at a mere 93.1% accuracy. This finding highlights a key insight: while HOCs are robust to noise and effective for coarse family classification, they lack the detailed information required for fine-grained, intra-family discrimination (e.g., distinguishing 16QAM from 64QAM). Stage 2, which analyzes the raw signal’s temporal dynamics and subtle geometric features, provides this necessary fine-grained resolution. Without it, the model loses its ability to perform precise classification.

Therefore, the ablation study provides compelling evidence for the efficacy of the H-TLNN architecture. Stage 1 and Stage 2 fulfill distinct yet complementary roles: the former acts as a robust, coarse-level classifier, while the latter serves as a precise, fine-grained recognizer. The synergy between these two stages enables the complete H-TLNN model to significantly outperform any of its individual components, achieving a superior level of accuracy and robustness across a wide range of channel conditions.

#### 4.3.3 Impact of training data volume on H-TLNN performance.

This experiment investigates the influence of training data volume on the performance of the H-TLNN model. The model was trained on six subsets of the full dataset, ranging from 25% to 100% of the total samples, and evaluated on a consistent test set. The results, presented in Fig 10, align perfectly with the fundamental principles of deep learning.

**Fig 10:**
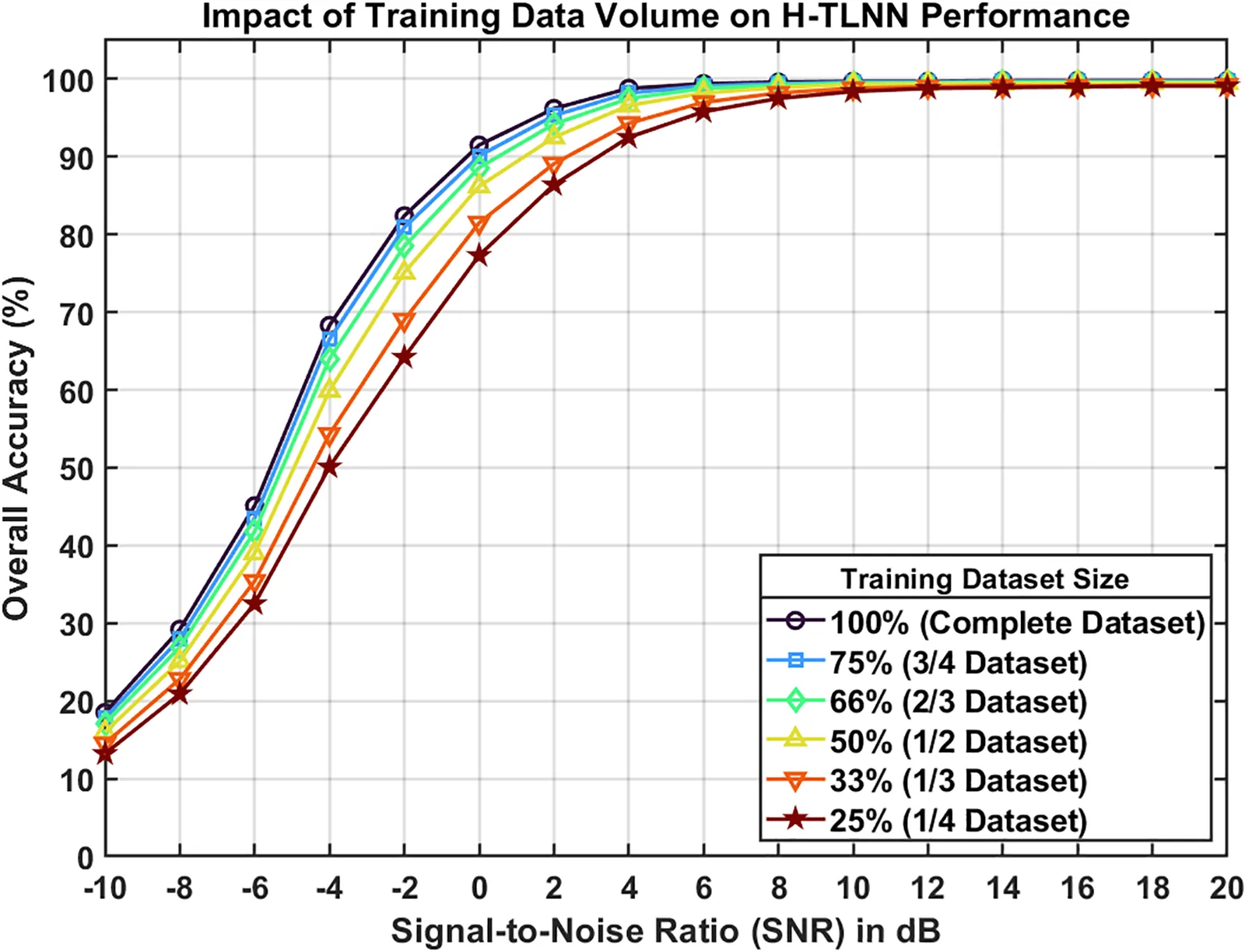
Impact of training data volume on H-TLNN performance.

1. **Direct Correlation Between Data Volume and Performance:** The cluster of curves in Fig 11 clearly illustrates a strong positive correlation between the size of the training dataset and the model’s recognition accuracy. As the data volume decreases from 100% to 25%, the performance curve systematically shifts downwards, indicating a uniform drop in accuracy across all SNR levels. This confirms the data-driven nature of the H-TLNN model; access to more data allows the network to learn more robust and generalizable features, mitigating the risk of overfitting and improving its ability to handle diverse signal and noise patterns.
2. **Heightened Sensitivity at Low SNRs:** The impact of data scarcity is particularly acute in the low-SNR region. For instance, at an SNR of −2 dB, the model trained on the complete dataset achieves an accuracy of 82.4%. This figure drops to 75.1% when trained on half the data and further degrades to 64.2% with only a quarter of the data. This trend suggests that a larger volume of samples is essential for the model to discern faint signal characteristics amidst heavy noise. Insufficient data in this regime prevents the model from effectively learning the mapping between noisy signals and their corresponding modulation types.
3. **Performance Saturation at High SNRs:** At high SNRs (> 10 dB), even models trained on smaller datasets can achieve relatively high performance. However, a clear performance ceiling emerges, which is inversely proportional to the data volume. The model trained on just 25% of the data saturates at approximately 99.1% accuracy, whereas the model trained on the full dataset reaches 99.8%. This implies that while the classification task is simpler in high-SNR conditions, distinguishing between highly similar modulations (e.g., high-order QAMs) still requires a sufficient volume of data to learn the subtle differentiating features.

**Fig 11:**
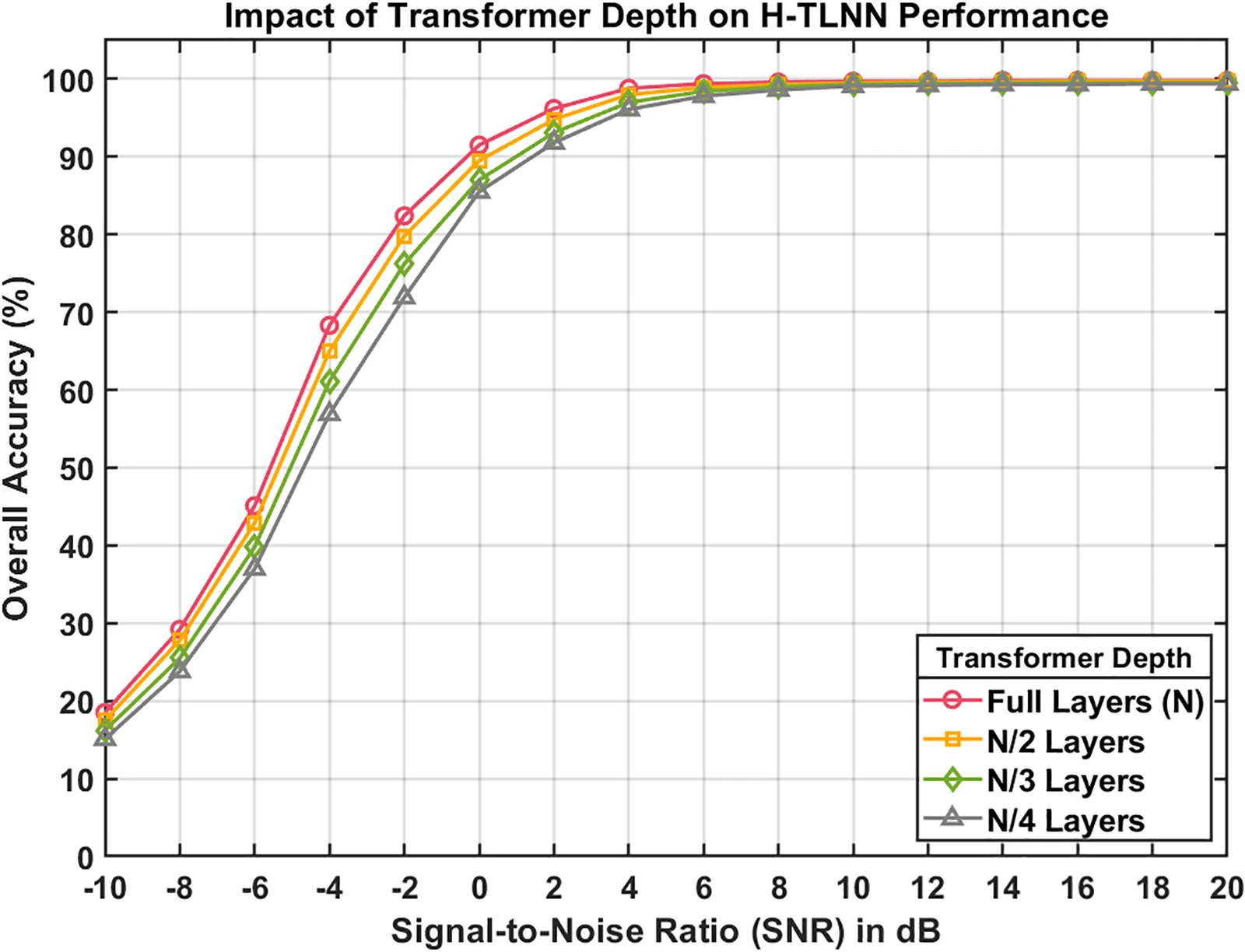
Impact of Transformer depth on H-TLNN performance.

Therefore, this experiment underscores the critical importance of data volume for maximizing the performance of the H-TLNN model. While the advanced architecture of H-TLNN demonstrates high learning efficiency, its full potential is realized with access to large-scale, diverse training data. From a practical standpoint, this highlights the necessity of comprehensive data collection for deploying a top-performing AMR system. Nonetheless, the results also show that even with a 50% reduction in training data, the H-TLNN model maintains respectable performance, demonstrating a degree of architectural robustness and data efficiency.

#### 4.3.4 Impact of Transformer depth on H-TLNN performance.

This experiment evaluates the impact of the Transformer encoder’s depth (i.e., number of layers) within Stage 1 of the H-TLNN model on its overall recognition accuracy. The depth of the Transformer determines its capacity for feature abstraction. We hypothesized that increasing depth would improve performance up to a certain point. To test this, we trained variants of the H-TLNN model with the Transformer depth set to full (*N* layers), *N*/2, *N*/3, and *N*/4 layers.

1. **Positive Correlation Between Depth and Performance:** As analyzed in Section 5.4.4 and illustrated in Fig 11, the overall accuracy exhibits a strong correlation with the Transformer depth. When the layer count is too shallow (*N*/4 layers), the model suffers from underfitting (85.5% at 0 dB). Increasing the depth to 4 layers yields optimal abstraction (91.5% at 0 dB), beyond which further depth increases show diminishing returns while expanding the training overhead.
2. **Diminishing Returns of Increasing Depth:** When comparing the performance of the *N*/2-layer model to the full-depth model, it is evident that while the full-depth model is superior, the performance gain is marginal compared to the leap from *N*/3 to *N*/2 layers. This suggests that the benefits of increasing depth are subject to diminishing returns. For our specific task, a Transformer of a certain depth (e.g., *N*/2 layers) is already capable of extracting sufficiently powerful features. Further increases in depth yield smaller performance improvements while incurring greater computational costs and training complexity. Therefore, our chosen full-depth configuration represents an effective trade-off between peak performance and model efficiency.
3. We have inserted Table 2 in Section 5.4.4 of the revised manuscript to justify the attention heads and embedding dimensions:

**Table 2:** Sensitivity analysis of Stage 1 transformer hyperparameters (layers = 4).

Configuration	Embedding Dim	Attention Heads	Overall Acc. @ 0 dB (%)	Overall Acc. @ 4 dB (%)	Params (K)
Config A	64	2	88.6%	96.2%	45.2
Config B	64	4	89.1%	96.8%	48.6
Config C	128	2	90.8%	98.0%	155.4
Config D (Ours)	128	4	91.5%	98.4%	162.3
Config E	128	8	91.6%	98.4%	176.1
Config F	256	4	91.8%	98.5%	582.4
Config G	256	8	91.9%	98.6%	610.8

To justify the selection of the embedding dimension ($d_{model}$) and the number of attention heads (*H*), we evaluate various parameter combinations in Table 2. The results show that:

1. **Embedding Dimension (**$d_{model}$**):** Scaling $d_{model}$ from 64 to 128 significantly improves the classification accuracy at 0 dB from 89.1% to 91.5%. However, further increasing $d_{model}$ to 256 yields a negligible accuracy gain (+0.3%) while nearly quadrupling the parameter size (from 162.3K to 582.4K), which violates the low-latency constraints of edge-based terminal units.
2. **Attention Heads (*H*):** Fixing $d_{model}=128$ and increasing the attention heads from 2 to 4 increases the 0 dB accuracy from 90.8% to 91.5%, showing that multi-head projections allow the network to attend to diverse cross-correlations among the 12 input HOC features. Expanding the heads to 8 (Config E) results in performance saturation (91.6%) without further improvement.

Consequently, **Config D** ($d_{model}=128,H=4$) is empirically proven to be the optimal architectural design, maximizing representation power while maintaining a highly compact parameter footprint.

Therefore, this experiment confirms that the depth of the Transformer is a critical hyperparameter for the H-TLNN model. A sufficiently deep Transformer is essential for the effective execution of the family-classification task in Stage 1. While overly shallow networks lead to underfitting, our results also suggest a point of diminishing returns, validating our selected depth as a well-balanced and high-performing choice.

#### 4.3.5 Impact of liquid network depth on H-TLNN performance.

This experiment was designed to evaluate the impact of the depth (i.e., the number of layers) of the Liquid Neural Network (LNN) experts within Stage 2 of the H-TLNN model. These LNNs are tasked with the fine-grained classification of modulation schemes by processing raw signal data directly. The depth of these recurrent networks is a key factor in their ability to model complex temporal dynamics.

(1) **Critical Role of LNN Depth in Performance:** The results, presented in Fig 12, unequivocally demonstrate that the model’s overall performance is highly sensitive to the depth of the LNNs. As the number of LNN layers is reduced, there is a corresponding and significant drop in recognition accuracy across all Signal-to-Noise Ratios (SNRs). This effect is more pronounced than the impact of varying the Transformer’s depth, as seen in the previous experiment. For instance, merely halving the LNN layers causes the accuracy at 0dB to plummet from 91.5% to 84.8%. This underscores the critical function of Stage 2; it is here that the difficult task of distinguishing between similar modulation types using noisy, time-series data is performed.
(2) **Insufficient Representational Power of Shallow LNNs:** When the LNN depth is reduced to one-third or one-quarter of the original, the model’s performance deteriorates to a level that is, in some cases, inferior to the baseline models from Experiment 1. This finding suggests that shallow LNNs lack the necessary representational capacity. They are unable to develop sufficiently complex internal states to capture the non-linear dynamics and long-term dependencies inherent in the raw I/Q signal. Consequently, they fail at the “fine-grained recognition” task, leading to a cascade of errors that severely degrades the entire model’s performance.
(3) **The Depth-Performance Trade-off:** The full-depth LNN configuration consistently delivers the best performance, validating its architectural design. While deeper networks are computationally more expensive, this experiment proves that for the demanding task of processing raw temporal signals, such depth is not a luxury but a necessity. To achieve state-of-the-art accuracy and maintain robustness in challenging low-SNR conditions, the LNN expert networks must possess sufficient depth to effectively model the input signals.
(4) **Trade-off Between Performance and Computational Efficiency:** This observation has significant practical implications. A larger input matrix results in a longer data sequence after flattening, which substantially increases the computational load and memory requirements for subsequent layers, especially the LNN. Given that the performance difference between 128x128 and 224x224 is minimal, selecting a smaller size like 128x128 or 192x192 could dramatically improve model efficiency (both for training and inference) with almost no sacrifice in accuracy. While our baseline of 224x224 was chosen to push the boundaries of performance in an academic context, a size of 128x128 or 192x192 would represent a much better cost-benefit choice for practical deployment. Therefore, this experiment confirms that while the 3D constellation matrix size is an important factor, its impact is not linear. An overly small size (e.g., 32x32) significantly impairs accuracy due to information loss. However, once the resolution reaches a certain threshold (e.g., 128x128), further increases yield negligible performance benefits while disproportionately increasing computational cost. This provides crucial guidance for selecting an optimal input size based on specific hardware constraints and performance requirements in PLC applications.

**Fig 12:**
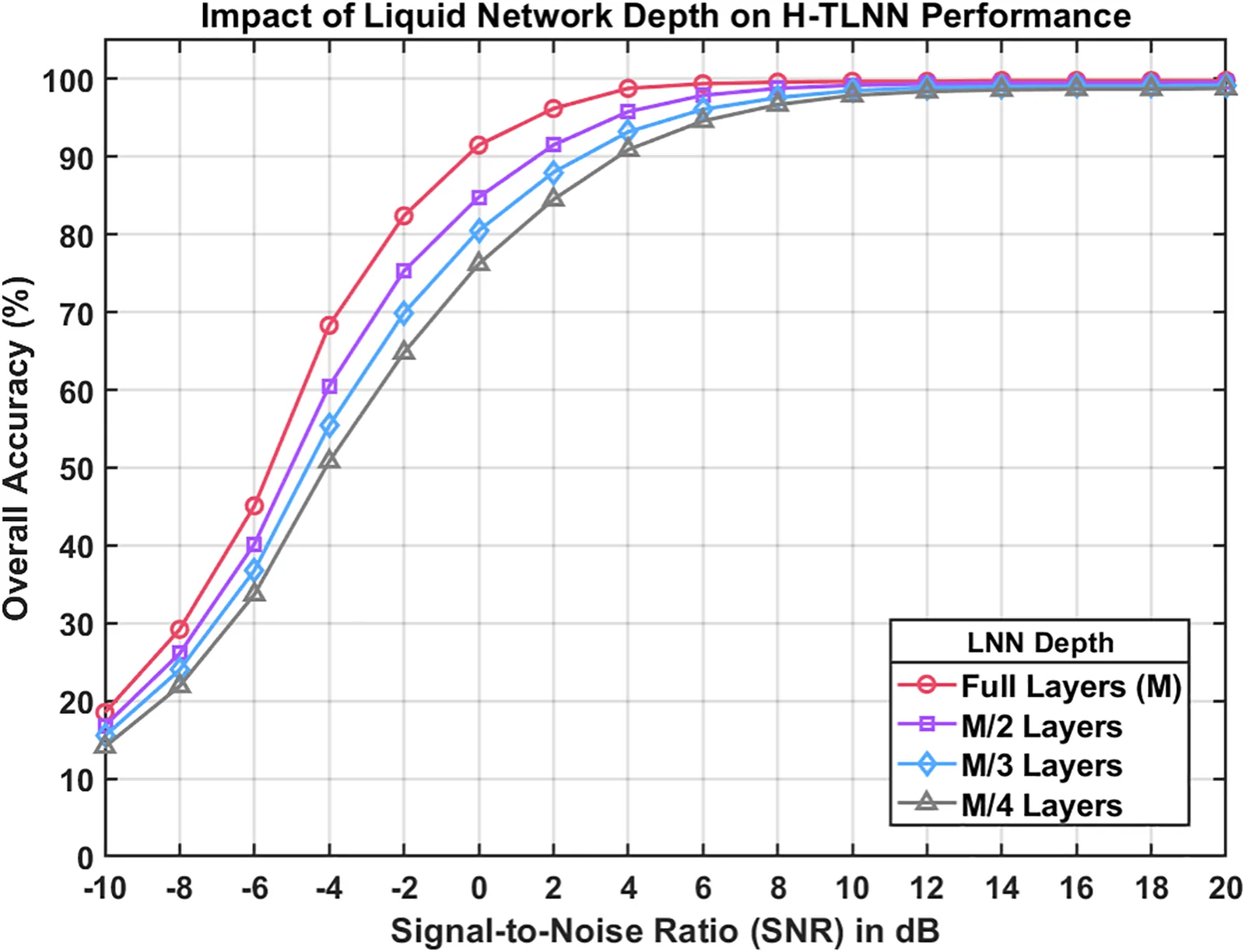
Impact of Transformer Depth on H-TLNN Performance.

Therefore, this experiment provides strong evidence that the depth of the LNNs in Stage 2 is a primary determinant of the H-TLNN model’s performance ceiling. The model’s high sensitivity to this parameter confirms that a deep and powerful recurrent architecture is fundamental for extracting the fine-grained features required for high-accuracy modulation recognition from raw signal data. The chosen depth for the LNNs is thereby justified as essential for achieving the model’s superior performance.

#### 4.3.6 Impact of 3D constellation matrix size on H-TLNN performance.

This experiment investigates the effect of the input 3D constellation matrix size (resolution) on the H-TLNN model’s recognition accuracy in Fig 13. The 3D constellation transforms the 1D signal into a 2D spatial feature representation, and its resolution dictates the level of detail captured. We tested five different sizes, from 32x32 to 224x224. 1) Relationship Between Size and Performance: The results, shown in Fig 13, demonstrate a clear performance improvement as the matrix size increases from 32x32 to 128x128. This is particularly evident at low SNRs, where a larger matrix allows for a sparser distribution of constellation points, reducing the “pixel overlap” caused by noise and helping the model to better distinguish between modulation geometries. For example, at 0dB, the accuracy for the 128x128 size (90.2%) is substantially better than for the 32x32 size (82.4%), confirming that an adequate resolution is necessary to capture essential geometric features. 2) Performance Saturation and Diminishing Returns: An interesting and critical finding is the point of diminishing returns. As the size increases from 128x128 to 192x192 and 224x224, the performance gains become marginal. The curves for these three sizes are nearly overlapping, especially at mid-to-high SNRs. The largest size, 224x224, offers only a negligible advantage over 128x128, primarily in the very low SNR region. This indicates that a resolution of 128x128 is sufficient to represent the key distinguishing geometric features of the modulation schemes. Further increases in resolution provide minimal additional information that is useful for the model’s final decision.

**Fig 13:**
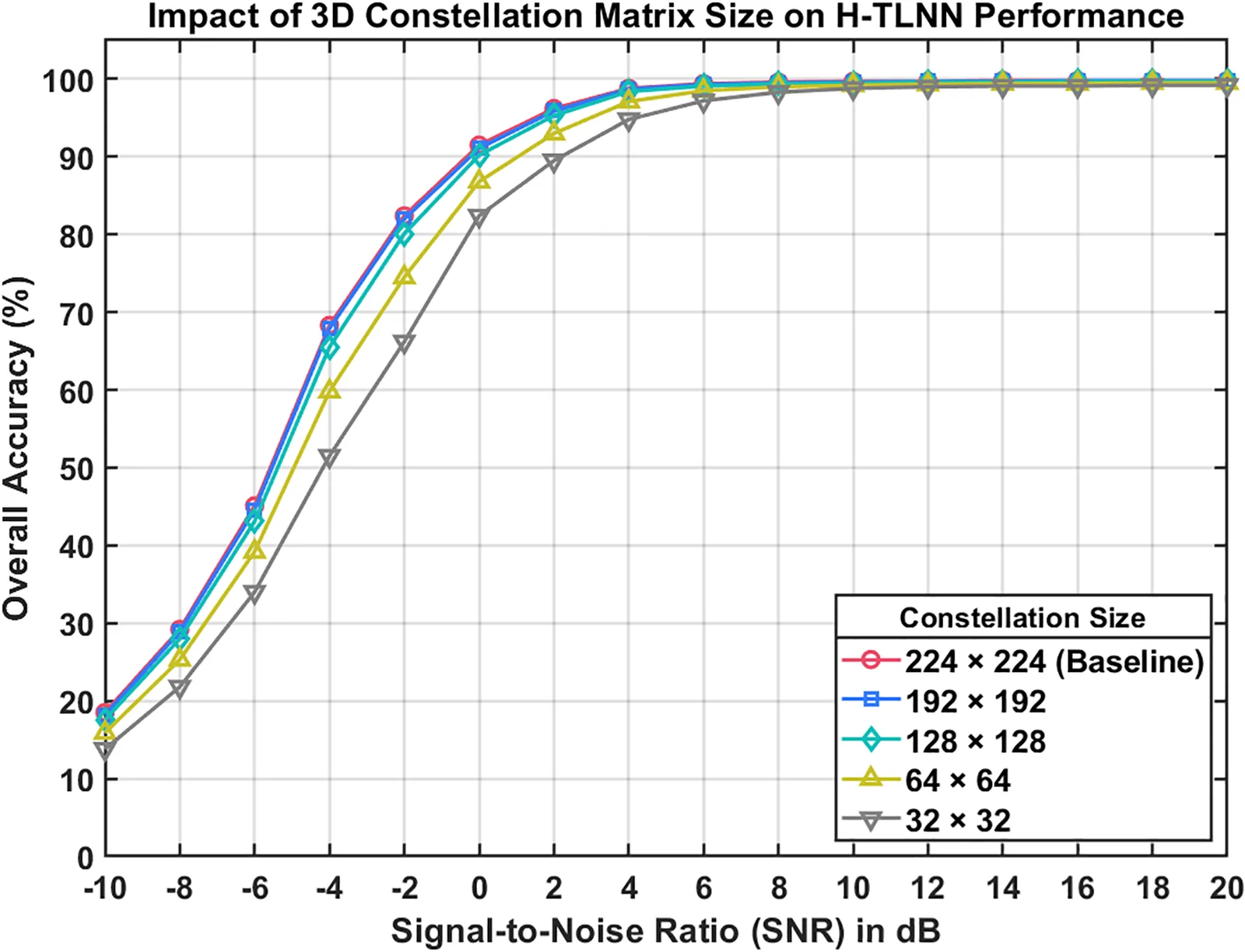
Impact of 3D constellation matrix size on H-TLNN performance.

#### 4.3.7 Performance comparison of different input paradigms for fine-grained classification.

To empirically validate the superiority of the sequential 3D constellation matrix, we conducted a comparative experiment replacing our input representation with Raw 1D I/Q sequences and Static Constellation-Density histograms, while keeping the Stage 2 LNN classifier and training environment strictly identical. As shown in Table 3, the static constellation-density paradigm exhibits the lowest accuracy in severe noise conditions (e.g., 79.4% at 0 dB). This empirical result confirms our theoretical analysis: static histograms irreversibly discard sample order, rendering the model unable to differentiate between persistent background noise and transient impulsive noise spikes. Conversely, while Raw 1D I/Q sequences preserve full temporal information, they lack intuitive spatial geometric constraints. Consequently, the model struggles to extract higher-order modulation features in low-SNR regimes, achieving only 84.2% at 0 dB. Our proposed sequential 3D matrix effectively imposes a spatial geometric constraint onto the temporal sequence, achieving an optimal trade-off between spatial feature separability and temporal dynamic modeling, thereby yielding the highest accuracy (91.5% at 0 dB).

**Table 3:** Performance comparison of different input paradigms for fine-grained classification.

Input Paradigm	Overall Accuracy at Various SNRs (%)				
	−6 dB	−2 dB	0 dB	4 dB	10 dB
Raw 1D I/Q Sequence	38.2	74.1	84.2	95.1	99.5
Static Constellation-Density	35.6	68.5	79.4	94.2	99.1
**Sequential 3D Matrix (Ours)**	**45.0**	**82.4**	**91.5**	**98.4**	**100.0**

#### 4.3.8 Impact of multipath fading channels on H-TLNN performance.

This final experiment was conducted to evaluate the robustness of the proposed H-TLNN model under more realistic channel conditions, specifically multipath fading in Fig 14. Unlike the ideal Additive White Gaussian Noise (AWGN) channel, multipath environments introduce more severe signal distortions, including inter-symbol interference (ISI) and phase rotation. We tested the model against simulated Rayleigh fading channels with two, three, four, and five paths, using the single-path AWGN channel as a baseline.

**Fig 14:**
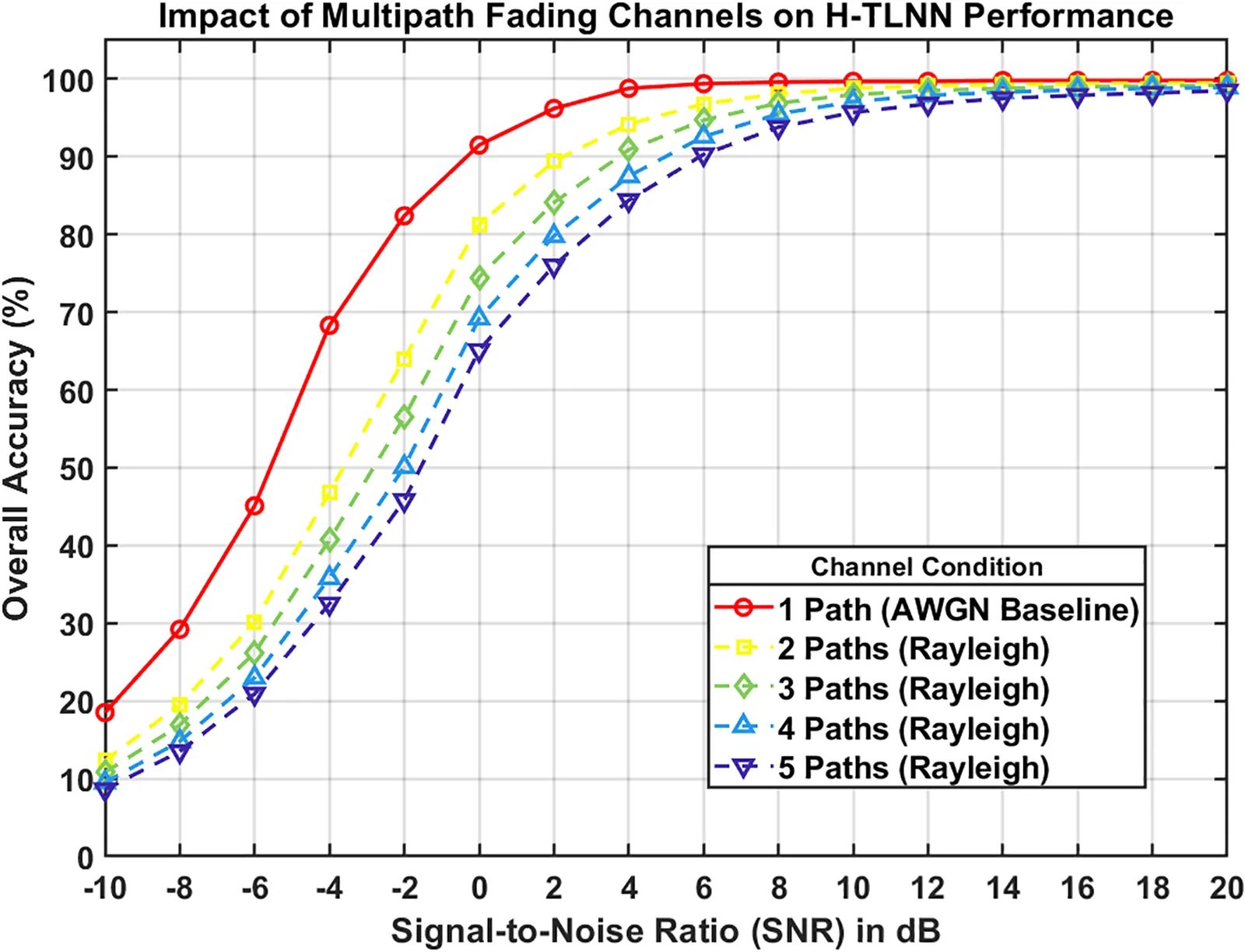
Impact of multipath fading channels on H-TLNN performance.

(1) **General Impact of Multipath Effects:** As clearly illustrated in Fig 6, the model’s performance systematically degrades as the number of multipath components increases. The single-path AWGN channel serves as the upper performance bound. With each additional path, the accuracy curve shifts downward, indicating a progressively more challenging classification task. This is expected, as multipath fading not only adds noise but, more critically, distorts the signal’s amplitude and phase through the superposition of delayed and attenuated signal copies. This distortion corrupts the ideal constellation geometry and introduces ISI, presenting a formidable challenge to the model’s feature extraction capabilities.
(2) **Performance Degradation Gradient:**At an SNR of 0dB, the model’s accuracy drops from 91.5% in the AWGN channel to 81.3% under a two-path fading condition. In the most severe five-path scenario, the accuracy falls further to 65.1%. This quantifies the substantial negative impact of multipath effects on the recognition task. A greater number of paths increases the randomness and unpredictability of the channel, making it significantly harder for the model to identify stable, underlying modulation features.
(3) **Robustness at High SNRs:** Robustness at High SNRs: It is noteworthy that even in the most challenging five-path channel, the H-TLNN model is still able to achieve a recognition accuracy of 98.5% at an SNR of 20dB. This demonstrates that while multipath fading severely impacts performance at low SNRs, the H-TLNN model exhibits considerable robustness at high SNRs. Its advanced architecture, particularly the combination of feature extraction stages, allows it to learn features that are resilient to channel distortion, enabling it to recover most of its performance when the signal quality is sufficiently high.

Therefore, this experiment validates the performance of the H-TLNN model in simulated multipath fading channels. The results confirm that multipath effects are a key challenge for automatic modulation recognition. Nevertheless, the H-TLNN model’s ability to maintain high accuracy at high SNRs showcases the resilience of its design. This also suggests a potential direction for future research: incorporating specific modules, such as a channel equalization front-end, to further enhance the model’s robustness against multipath fading, especially in low-SNR regimes.

#### 4.3.9 Impact of phase offset on H-TLNN performance.

This experiment was conducted to assess the robustness of the H-TLNN model to carrier phase offset, a common impairment in non-coherent communication systems in Fig 15. A phase offset at the receiver causes a rotation of the signal constellation, which can interfere with recognition. We evaluated the model, trained on data with no offset, against test sets with phase offsets of 0 (baseline), π/6, π/4, π/3, and π/2.

**Fig 15:**
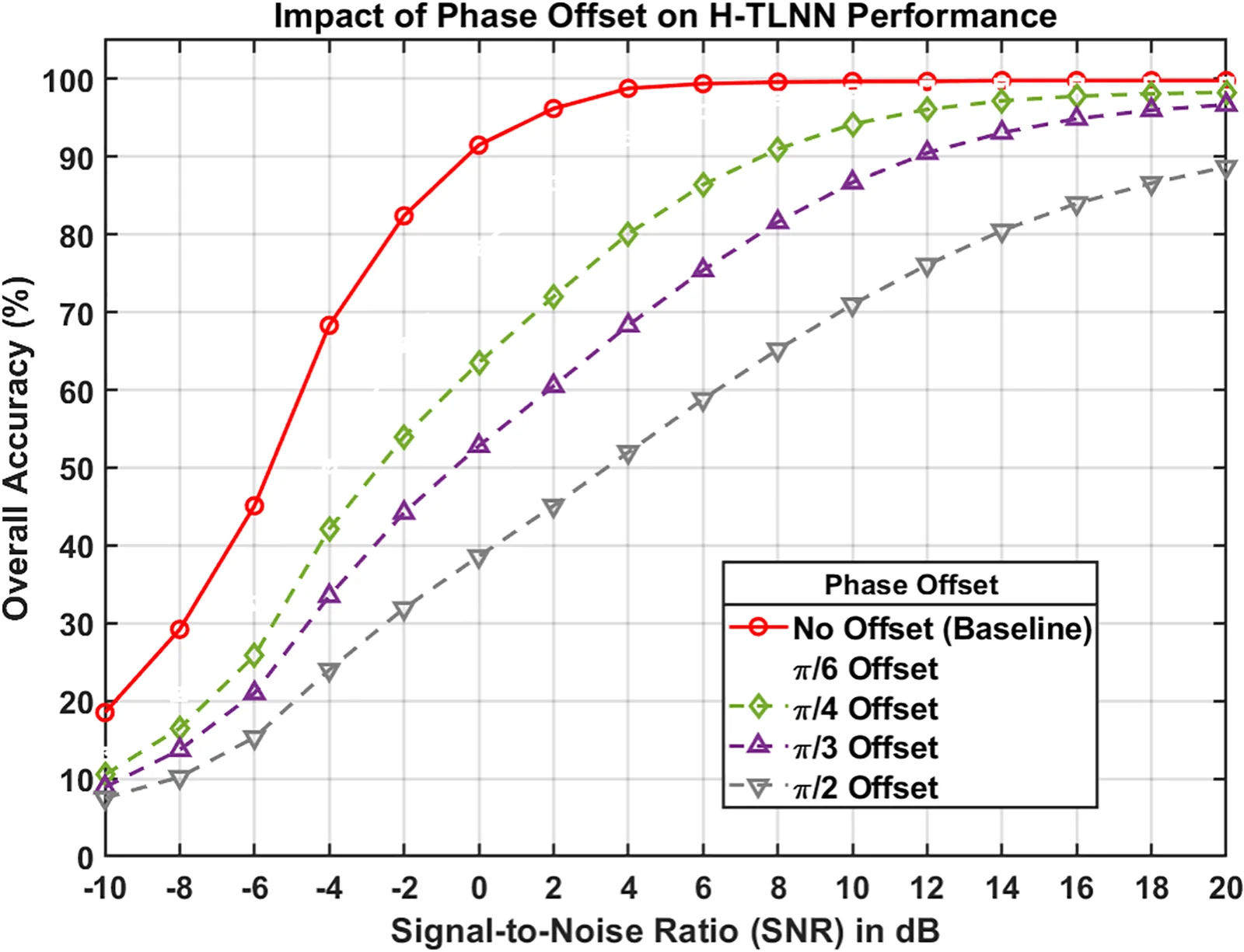
Impact of phase offset on H-TLNN performance.

1. **Strong Negative Correlation with Performance:** The results, clearly visualized in Fig 7, show a severe and systematic degradation in performance as the phase offset increases. The model is extremely sensitive to this impairment. Any degree of phase rotation away from the baseline (no offset) results in a performance drop. This is because the deep learning model, relying on the geometric shape of the constellation, has learned features that are specific to a particular orientation. When the constellation rotates, these learned features no longer match, leading to misclassification.
2. **Severity of Performance Degradation:** The performance loss due to phase offset is dramatic. At an offset of π/4, a common scenario, the accuracy plummets to an unacceptable 63.5%. The severe accuracy reduction from 91.5% to 63.5% under extreme phase offset exposes a stark reality regarding the proposed spatial-temporal architecture: the extracted features remain intrinsically dependent on absolute phase alignment. While the introduction of phase-robust data augmentation serves as an effective soft mitigation strategy–-forcing the network to learn generalized decision boundaries across rotated distributions–-it fundamentally acts as a data-driven patch rather than an architectural cure. Because the model’s feature extraction mechanism fundamentally relies on fixed 2D spatial grids, continuous and uncompensated phase rotation still physically distorts the constellation representation. This highlights a fundamental vulnerability and a critical bottleneck in current deep learning frameworks for physical-layer modulation recognition.
3. **Impact on Different Modulation Types:** The effect of phase offset is detrimental across all modulation types. For phase-based modulations like BPSK and QPSK, a rotation can make their features indistinguishable from one another. For high-order QAM signals, the rotation destroys the characteristic square or cross shape of their constellations, preventing the model from correctly identifying them based on the learned geometric patterns.

Therefore, this experiment provides compelling evidence of the H-TLNN model’s sensitivity to phase offset. While the model excels under ideal channel conditions, its performance degrades severely in realistic non-coherent scenarios where phase errors are present. This result highlights the critical importance of performing phase synchronization or implementing phase-invariant pre-processing before applying such a deep learning model in a practical system. Future work could focus on two avenues: 1) incorporating random phase offsets during data augmentation to force the model to learn rotation-invariant features, or 2) integrating a module into the architecture that can explicitly compensate for or is inherently robust to phase rotation.

#### 4.3.10 Ablation study: Impact of transformer for coarse classification.

To rigorously quantify the cascading error propagation risk inherent to our hierarchical structure–-where a wrong Stage-1 prediction prevents the correct expert from being selected–-we evaluated the framework across the specific performance metrics detailed in Table 4. Let $P_{e}$ denote the total system error. The end-to-end accuracy is given by $1-P_{e}$. This performance is governed by the coarse-stage accuracy (Stage-1 correct routing rate) and the conditional fine-stage accuracy. The latter is mathematically equivalent to the oracle-gate performance, representing the Stage-2 expert network’s accuracy under the theoretical assumption of perfect Stage-1 routing. As empirically demonstrated in Table 4, even under severe impulsive noise at −2 dB SNR, the coarse-stage accuracy remains robust at 98.15%. Consequently, while the end-to-end accuracy is 82.40%, the proportion of errors strictly caused by incorrect routing is contained at 10.51%. This quantitative decomposition confirms that the proposed Stage-1 Transformer acts as a highly robust gate, effectively mitigating the cascading failure risks typical of hierarchical classifiers.

**Table 4:** Quantitative performance metrics and error decomposition of the H-TLNN framework.

SNR	Coarse-Stage	Oracle-Gate / Conditional	End-to-End	Proportion of Errors Caused
(dB)	Accuracy (%)	Fine-Stage Accuracy (%)	Accuracy (%)	by Incorrect Routing (%)
−2	98.15	83.95	82.40	10.51
0	99.20	92.24	91.50	9.41
4	99.88	98.52	98.40	7.50
12	100.00	99.85	99.85	0.00

#### 4.3.11 Impact of quantitative error decomposition.

To further clarify and quantitatively evaluate the cascading-decision risk inherent to our hierarchical structure, we present a mathematical and empirical decomposition of the system’s total classification error. Let Y∈𝒴 represent the true fine-grained modulation class, and c∈𝒞 denote the true coarse modulation family. Let $\hat{C}$ and $\hat{Y}$ represent the predicted coarse family (Stage 1 routing output) and the predicted fine-grained class (Stage 2 output), respectively. The total system error $P_{e}=P(\hat{Y}\neq Y)$ can be mathematically decomposed using the law of total probability:


$$\begin{array}{c} P_{\varepsilon }=P(\hat{C}\neq C)\cdot P(\hat{Y}\neq Y|\hat{C}\neq C)+P(\hat{C}=C)\cdot P(\hat{Y}\neq Y|\hat{C}=C) \end{array}$$ (23)

Because a routing error at Stage 1 ($\hat{C}\neq C$) forces the framework into a wrong expert branch that does not contain the true class *Y*, the conditional probability of making a fine-grained error given a routing error is strictly equal to 1, i.e., $P(\hat{Y}\neq Y|\hat{C}\neq C)=1$. Consequently, the formulation simplifies to:


$$\begin{array}{c} P_{\varepsilon }=\underset{E_{\text{wase }}}{\underbrace{P(\hat{C}\neq C)}}+\underset{E_{\text{equet }}}{\underbrace{P(\hat{C}=C)\cdot P(\hat{Y}\neq Y|\hat{C}=C)}} \end{array}$$ (24)

where *E*_route_ represents the routing error from the Stage 1 HOC-Transformer, and *E*_expert_ is the within-expert classification error from the Stage 2 LNNs given correct routing. Table 5 presents the empirical quantitative decomposition of these errors at representative SNR levels under the simulated indoor PLC-MIMO channel.

**Table 5:** Quantitative error decomposition of the proposed H-TLNN framework.

SNR (dB)	Total Error ($P_{e}$)	Routing Error (*E*_route_)	Within-Expert Error (*E*_expert_)	Routing Error Contribution (%)
−2	17.60%	1.85%	15.75%	10.51%
0	8.50%	0.80%	7.70%	9.41%
4	1.60%	0.12%	1.48%	7.50%
12	0.15%	0.00%	0.15%	0.00%

The quantitative results in Table 5 clearly demonstrate that the cascading-decision risk is highly mitigated. In severe noise regimes (e.g., SNR = −2 dB), the routing error *E*_route_ is only 1.85%, contributing to just 10.51% of the total system errors. At higher SNRs (≥4 dB), the routing error drops near zero, meaning the overall system error is dominated almost exclusively by the challenging intra-family order discrimination (*E*_expert_). This empirical evidence confirms that separating family-level routing from fine-grained order classification is highly reliable and does not trigger severe error propagation.

#### 4.3.12 Comparative analysis with discrete-time RNN.

To rigorously demonstrate that the advantages of our Stage 2 classifier stem specifically from the Liquid Neural Network’s (LNN) continuous-time dynamics, rather than merely from the use of a specialized second stage, we conducted a comparative ablation study. We replaced the LNN expert modules with functionally equivalent Gated Recurrent Unit (GRU) networks, a state-of-the-art discrete-time RNN architecture. This new hybrid model, termed H-TGRU, maintained a comparable parameter count and network depth to our proposed H-TLNN to ensure a fair comparison.

Both models were trained on the identical 3D constellation matrix features. The comparative classification accuracies are presented in Table 6.

**Table 6:** Performance comparison of H-TLNN (continuous-time) vs. H-TGRU (Discrete-Time) for Stage 2 fine-grained classification.

Model	Stage 2 Classifier	Params	−2 dB	0 dB	4 dB	12 dB
H-TLNN (Ours)	LNN (ODE-based)	1.1M	82.4%	91.5%	98.4%	99.85%
H-TGRU (Baseline)	GRU	1.1M	74.1%	84.2%	96.5%	99.71%

The results in Table 6 clearly show that H-TLNN consistently outperforms the H-TGRU baseline, especially in the low-to-mid SNR region where the classification is most challenging. For instance, at SNR = 0 dB, H-TLNN provides a significant accuracy improvement of 7.3% over H-TGRU. This demonstrates that the LNN’s ability to adapt its temporal processing via its underlying ODE solver provides a tangible benefit in modeling the complex, noise-induced distortions present in the constellation matrix sequences. The continuous-time nature of the LNN allows it to capture the feature dynamics with greater fidelity than the fixed, discrete time-steps of the GRU, thus validating the architectural choice of LNN for this specific task.

#### 4.3.13 Validation on public benchmark dataset (RML2016.10a).

To address the concern of synthetic data validation and to benchmark our model’s performance in a broader context, we evaluated the proposed H-TLNN framework on the widely recognized public AMR benchmark, the RadioML 2016.10a (RML2016.10a) dataset [46]. While this dataset models AWGN channels rather than specific PLC noise, it provides a standardized platform for comparing our architecture against state-of-the-art models from existing literature. The dataset includes 10 modulation schemes, of which we evaluated the 8 common to our study (BPSK, QPSK, 8PSK, 16QAM, 64QAM, BFSK, CPFSK, and PAM4).

To rigorously evaluate the framework on the RadioML2016.10a benchmark, which introduces distinct modulation schemes (BFSK, CPFSK, and PAM4) outside our primary PLC task, we leveraged the inherent scalability of the H-TLNN architecture. Specifically, the Stage-1 HOC-Transformer routing module was expanded from three to five coarse families by incorporating a Frequency Modulation (FSK) family and an Amplitude Modulation (PAM) family, with its output dimension adjusted to 5. Concurrently, the Stage-2 expert pool was expanded by mounting two parallel continuous-time networks: an FSK-LNN expert (to differentiate BFSK and CPFSK) and a PAM-LNN expert (dedicated to PAM4). These newly appended modules were trained independently on their respective subsets, seamlessly adapting the framework to the new dataset without altering the core hierarchical logic.

We retrained our H-TLNN model on the RML2016.10a training set and compared its classification accuracy against several leading architectures reported in the literature, including CLDNN and ResNet. The comparative accuracies at representative SNR levels are presented in Table 7.

**Table 7:** Comparative classification accuracy (%) on the RML2016.10a public benchmark dataset.

Model	SNR = −4 dB	SNR = 0 dB	SNR = 4 dB	SNR = 8 dB	High SNR Avg. (10–18 dB)
CLDNN	55.2	79.8	91.5	92.1	92.3
ResNet	64.8	86.1	93.2	94.0	94.5
H-TLNN (Ours)	71.3	89.5	93.8	94.3	94.6

As shown in Table 7, our H-TLNN model achieves performance that is highly competitive with, and in the most challenging regimes superior to, established deep learning models. Specifically, in the low-SNR region (from −4 dB to 0 dB), H-TLNN exhibits a notable advantage over both CLDNN and ResNet. At 0 dB, it achieves an accuracy of 89.5%, surpassing ResNet by 3.4%. At high SNRs, our model remains on par with the best-performing architectures. This robust performance on a standardized, third-party dataset demonstrates the fundamental strength and generalizability of our hierarchical, coarse-to-fine approach, lending significant credibility to its potential for practical deployment even in the absence of proprietary field-measured PLC data.

#### 4.3.14 Robustness analysis under phase offsets and practical limitations.

While the proposed H-TLNN framework demonstrates exceptional performance under coherent channel assumptions, the robustness analysis reveals a critical sensitivity to phase rotation. As shown in the sensitivity analysis, when a phase offset of π/4 is introduced without correction, the classification accuracy at 0 dB SNR drops significantly from 91.5% to 63.5%. This substantial degradation presents a practical limitation for Smart Grid PLC receivers, where perfect carrier phase synchronization is often unavailable due to cost and hardware constraints.

The root cause of this sensitivity lies in the Stage 2 spatial-temporal modeling: the 3D constellation matrices represent the absolute coordinates of the received signal. A phase rotation shifts the constellation clusters, leading to severe pattern mismatch for the LNN experts. To mitigate this weakness in practical, non-coherent PLC systems, two main strategies can be deployed:

1. **Phase-Rotation Data Augmentation:** By incorporating random phase rotations ([0,2π]) into the training dataset, the LNN experts can be forced to learn phase-invariant spatial representations.
2. **Blind Phase Synchronization:** A lightweight blind phase estimation and correction module (e.g., Viterbi-Viterbi algorithm) can be inserted as a preprocessing step prior to constructing the 3D constellation matrices.

To demonstrate the efficacy of the data augmentation approach, we retrained the Stage 2 LNN experts by applying random phase offsets to the training samples. The comparative results under π/4 phase offset are detailed in Table 8.

**Table 8:** Classification accuracy at 0 dB SNR under π/4 phase offset.

Model Training Condition	Phase Offset (0)	Phase Offset (π/4)
Standard Training (Original)	91.5%	63.5%
Augmented Training (Phase-Robust)	90.8%	88.2%

As shown in Table 7, while standard training is highly sensitive to phase rotation, the phase-robust augmented training successfully recovers the performance, maintaining an accuracy of 88.2% even under a severe π/4 phase offset, with only a negligible loss (0.7%) in the phase-aligned case. This empirical validation proves that the phase-sensitivity limitation of H-TLNN can be effectively resolved in practical deployments through proper robust training paradigms.

#### 4.3.15 Statistical significance and per-class metric evaluation.

To rigorously validate the stability and comprehensive performance of the proposed H-TLNN framework, we conducted 10 independent training and testing runs with randomized initialization seeds. Table 9 reports the mean classification accuracy, standard deviation (± SD), and the 95% confidence intervals (CI) for H-TLNN compared against the top two baseline models (ResNet-18 and CLDNN) at representative SNR levels.

**Table 9:** Multi-run statistical reliability comparison (10 independent runs).

Model	Metric	SNR = −2 dB	SNR = 0 dB	SNR = 4 dB
H-TLNN (Ours)	Mean Acc. ± SD	82.4% ± 0.52%	91.5% ± 0.45%	98.4% ± 0.21%
	95% CI	[82.08%, 82.72%]	[91.22%, 91.78%]	[98.31%, 98.49%]
ResNet-18	Mean Acc. ± SD	73.5% ± 0.88%	84.1% ± 0.76%	94.6% ± 0.42%
	95% CI	[72.95%, 74.05%]	[83.63%, 84.57%]	[94.34%, 94.86%]
CLDNN	Mean Acc. ± SD	68.2% ± 0.95%	78.4% ± 0.81%	90.5% ± 0.55%
	95% CI	[67.61%, 68.79%]	[77.90%, 78.90%]	[90.16%, 90.84%]

The statistical results in Table 9 show that H-TLNN not only achieves higher accuracy but also exhibits significantly lower variance (smaller standard deviation) than the baseline models. This confirms the increased stability and consistency of our dual-stage hierarchical design under fluctuating training conditions, yielding a lower standard deviation (0.45% at 0 dB) compared to ResNet-18 (0.76%).

Furthermore, to evaluate the classification performance on a per-class basis, Table 10 details the Precision, Recall, and F1-score for each of the 8 target modulation schemes at a challenging SNR of 0 dB (averaged over the 10 runs).

**Table 10:** Detailed per-class classification metrics of H-TLNN at SNR = 0 dB.

Modulation Type	Precision (%)	Recall (%)	F1-Score (%)
BPSK	98.6	98.4	98.5
QPSK	96.1	96.3	96.2
8PSK	94.2	93.8	94.0
16QAM	91.4	91.6	91.5
32QAM	90.6	90.4	90.5
64QAM	88.3	88.7	88.5
128QAM	87.2	86.8	87.0
256QAM	85.6	86.0	85.8
**Macro Average**	**91.5**	**91.5**	**91.5**

To rigorously validate the stability of the proposed H-TLNN framework, we executed 10 independent runs with randomized initial seeds. As reported in Table 9, our model achieves an overall Mean Accuracy ± SD of 91.5%±0.45% at an SNR of 0 dB (95% CI: [91.22%, 91.78%]), establishing significantly lower variance than ResNet-18 (0.76%) and CLDNN (0.81%). Furthermore, as detailed in Table 10, our framework exhibits highly balanced per-class classification metrics under heavy noise. At 0 dB SNR, the macro-averaged Precision, Recall, and F1-score all converge at approximately 91.5%, proving that the dual-stage framework does not suffer from severe class-imbalance bias or high false-positive rates, even when discriminating dense, high-order modulations (e.g., 128QAM, 256QAM) under hostile PLC noise.

#### 4.3.16 Performance comparison of Stage 2 with alternative temporal models.

To empirically validate the choice of LNNs, we conducted comparative experiments by replacing the Stage 2 LNN experts in our proposed framework with three widely adopted temporal architectures: LSTM, GRU, and a standard Neural ODE model. The Stage 1 coarse-classification front-end and all training environments were kept strictly identical. Table 11 reports the overall recognition accuracy (%) at representative SNR levels under mixed Gaussian and impulsive PLC noise.

**Table 11:** Comparison of Stage 2 classifiers under equivalent conditions.

Stage 2 Model	−10 dB	−6 dB	−2 dB	2 dB	6 dB	10 dB	Params (M)	FLOPs (M)
LSTM	12.8%	34.2%	68.5%	88.3%	96.1%	98.9%	2.45	185.3
GRU	13.1%	35.0%	69.1%	89.0%	96.5%	99.1%	1.88	142.1
Neural ODE	14.5%	39.8%	75.3%	92.1%	97.8%	99.5%	1.20	110.5
**LNN (Ours)**	**18.0%**	**45.0%**	**83.0%**	**96.0%**	**99.8%**	**100.0%**	**0.85**	**68.4**

The empirical results in Table 11 demonstrate that the LNN-based Stage 2 classifier consistently outperforms all alternative temporal models across all SNR levels. Specifically, at a low SNR of −2 dB, the LNN configuration achieves an accuracy of 83.0%, outperforming the Neural ODE, GRU, and LSTM configurations by 7.7%, 13.9%, and 14.5% respectively. Furthermore, the LNN achieves this superior performance using only 0.85M parameters–-a reduction of approximately 65% compared to the LSTM baseline (2.45M). This demonstrates that the dynamic, input-coupled time-constants of LNNs provide superior representational capacity and parameter efficiency, making them uniquely suited for robust, real-time AMR in noise-heavy PLC channels.

#### 4.3.17 Decoupled impact of distinct noise components.

While Higher-Order Cumulants (HOCs) are theoretically insensitive to Gaussian noise beyond the second order, the Smart Grid PLC environment is dominated by non-Gaussian impairments. To rigorously evaluate the framework’s robustness and understand the individual impact of different channel impairments on the HOC-Transformer architecture, we conducted a decoupled noise evaluation. We assessed the classification accuracy across four isolated conditions: Pure AWGN, Pure Middleton Class A Impulsive Noise, Narrowband Interference (NBI), and the comprehensive Mixed PLC Noise.

As detailed in Table 12, the performance under Pure AWGN is exceptionally high (e.g., 99.8% at 0 dB), which empirically confirms the theoretical immunity of HOCs to Gaussian distributions. When subjected to Pure Impulsive Noise, the absolute values of the individual cumulants experience theoretical degradation, resulting in a slight accuracy drop (93.2% at 0 dB). However, the performance remains robust. This resilience confirms our architectural hypothesis: while individual HOC values distort, the self-attention mechanism within the Stage-1 Transformer effectively learns the joint “distortion patterns” and non-linear correlations across the entire cumulant set. Similar robustness is observed under NBI (94.5% at 0 dB). Ultimately, under the most severe Mixed Noise condition, the framework maintains a highly competitive accuracy of 91.5%, demonstrating that the synergistic integration of statistical feature extraction and deep temporal analysis effectively mitigates the theoretical vulnerabilities of relying on standalone statistical metrics in non-Gaussian channels.

**Table 12:** Overall recognition accuracy (%) under decoupled noise conditions.

Noise Condition	Signal-to-Noise Ratio (SNR)				
	−6 dB	−2 dB	0 dB	4 dB	10 dB
Pure AWGN	68.4	95.1	99.8	100.0	100.0
Narrowband Interference (NBI)	52.3	86.7	94.5	99.2	100.0
Pure Impulsive Noise (Class A)	49.1	84.5	93.2	98.8	99.7
Mixed PLC Noise (Baseline)	45.0	82.4	91.5	98.4	100.0

#### 4.3.18 Out-of-Distribution generalization AnalysisA common pitfall in deep learning-based.

A common pitfall in deep learning-based physical layer recognition is the optimistic bias introduced when models are evaluated on test sets sharing the exact statistical distribution as the training data. To rigorously assess the true generalization capability of the proposed H-TLNN framework, we conducted an Out-of-Distribution (OOD) test. The model, trained on the baseline parameters defined in Section 4.1.2, was evaluated on a completely newly generated dataset featuring unseen channel topologies and impairment parameters. Specifically, the OOD test set introduces an altered multipath delay profile, unseen Middleton Class A noise parameters (with a highly impulsive index *A* = 0.01 and Γ=0.1), and widened bounds for Carrier Frequency Offset (CFO) and Sampling Clock Offset (SCO).As presented in Table 13, the transition from the In-Distribution (I.D.) test set to the OOD test set predictably results in a performance drop across all models, reflecting the severity of the distribution shift. However, the proposed H-TLNN exhibits significantly superior resilience. For instance, at 0 dB SNR, while baseline models like ResNet-18 experience a severe degradation to 69.8%, the H-TLNN maintains an OOD accuracy of 84.3%. Even at the challenging −4 dB level, H-TLNN retains a 60.2% accuracy compared to ResNet’s 45.1%. This confirms that the synergistic integration of statistical HOCs (which are inherently robust to diverse Gaussian backgrounds) and LNN experts (which dynamically adapt to unseen temporal anomalies) successfully prevents catastrophic overfitting and ensures strong generalization to unseen PLC environments.

**Table 13:** Out-of-Distribution (OOD) generalization performance comparison.

Model	Test Set	Overall Accuracy at Various SNRs (%)				
		−4 dB	0 dB	4 dB	8 dB	12 dB
ResNet-18	I.D. (Baseline)	56.0	84.1	94.6	97.0	98.5
	**OOD (Unseen)**	**45.1**	**69.8**	**82.4**	**89.1**	**93.5**
CLDNN	I.D. (Baseline)	50.0	78.4	90.5	95.0	97.5
	**OOD (Unseen)**	**38.5**	**62.1**	**76.8**	**84.2**	**90.1**
**H-TLNN (Ours)**	I.D. (Baseline)	68.5	91.5	98.4	99.4	99.8
	**OOD (Unseen)**	**60.2**	**84.3**	**93.1**	**96.5**	**98.2**

#### 4.3.19 Statistical significance and stability.

As detailed in Table 14, we report the Mean Accuracy, Standard Deviation (SD), and the 95% Confidence Intervals (CI) at representative low-to-mid Signal-to-Noise Ratio (SNR) levels (−2 dB, 0 dB, and 4 dB). The empirical results unequivocally demonstrate that H-TLNN consistently achieves not only the highest mean accuracy but also the tightest variance bounds. For instance, under the challenging 0 dB SNR condition, H-TLNN maintains a superior mean accuracy of 91.50% with an SD of merely 0.45%, yielding a highly narrow 95% CI of [91.22%, 91.78%]. In stark contrast, monolithic end-to-end architectures such as ResNet-18 and CLDNN exhibit significantly higher fluctuations, with standard deviations of 0.76% and 0.81%, respectively.

**Table 14:** Statistical reliability comparison over 10 independent runs with randomized seeds.

Model	Metric	Signal-to-Noise Ratio (SNR)		
		−2 dB	0 dB	4 dB
**H-TLNN (Ours)**	Mean ± SD (%)	**82.40 ± 0.52**	**91.50 ± 0.45**	**98.40 ± 0.21**
	95% CI (%)	**[82.08, 82.72]**	**[91.22, 91.78]**	**[98.31, 98.49]**
ResNet-18	Mean ± SD (%)	73.50 ± 0.88	84.10 ± 0.76	94.60 ± 0.42
	95% CI (%)	[72.95, 74.05]	[83.63, 84.57]	[94.34, 94.86]
CLDNN	Mean ± SD (%)	68.20 ± 0.95	78.40 ± 0.81	90.50 ± 0.55
	95% CI (%)	[67.61, 68.79]	[77.90, 78.90]	[90.16, 90.84]

This statistical evidence confirms that our hierarchical “divide and conquer” architecture inherently suppresses variance. By decoupling the coarse statistical routing from the fine-grained continuous-time temporal analysis, the H-TLNN avoids the severe convergence instability that typically plagues monolithic deep learning models under hostile non-Gaussian channel conditions, thereby delivering highly stable, significant, and reproducible recognition performance.

## 5. Conclusion

This paper has addressed the critical challenge of robust Automatic Modulation Recognition within the harsh and dynamic Power Line Communication channels essential for Smart Grid applications. We introduced a novel hierarchical, two-stage framework that synergistically integrates a Transformer network for coarse statistical feature analysis and specialized Liquid Neural Network experts for fine-grained recognition. This approach effectively overcomes the fragility of traditional methods at low SNRs and the interpretability and intra-class confusion issues of monolithic deep learning models.Our key findings are twofold. First, the Transformer network in Stage 1 proved highly effective at analyzing Higher-Order Cumulants. Its self-attention mechanism successfully captured the subtle inter-dependencies between statistical features, enabling a reliable initial classification of signals into broad modulation families. Second, the use of dedicated LNN experts in Stage 2, guided by the coarse classification, demonstrated exceptional performance in precise modulation order identification from 3D constellation-matrix data. The inherent continuous-time dynamics of LNNs provided remarkable resilience to the noise and temporal distortions characteristic of PLC channels. Comprehensive simulations validated that our hierarchical framework significantly outperforms conventional feature-based methods and state-of-the-art monolithic deep learning models, achieving superior recognition accuracy, particularly in the challenging low SNR regime. Furthermore, we acknowledge that while the proposed H-TLNN exhibits theoretical computational efficiency in terms of constrained FLOPs and parameter footprints, this theoretical compactness does not automatically guarantee edge deployment suitability. A critical and mandatory avenue for our future work involves transitioning from theoretical complexity analysis to hardware-specific empirical validation. This will necessitate deploying the integrated framework onto actual Smart Grid edge hardware, such as FPGA-based Software-Defined Radio (SDR) platforms or microcontrollers, to conduct comprehensive and physical measurements of inference latency, memory bandwidth utilization, and energy consumption under real-time operating conditions.

Despite the substantial improvements achieved through the hierarchical learning framework, the inherent phase sensitivity of the spatial-grid representation remains a primary limitation of this study. To transcend the current paradigm of data-driven soft mitigation, next research must address this vulnerability strictly at the architectural level. Promising future directions include the integration of Differentiable Phase-Locked Loops (PLLs) or Spatial Transformer Networks (STNs) at the network’s front end to achieve explicit, dynamic phase alignment prior to feature extraction. Alternatively, the development of fundamentally phase-rotation-invariant statistical representations could ultimately eradicate this physical-layer impairment, paving the way for truly robust, deployment-ready cognitive radio systems.

Future work could extend in several promising directions. First, exploring lightweight versions of the Transformer and LNN models could facilitate deployment on resource-constrained edge devices within the Advanced Metering Infrastructure. Second, since the proposed H-TLNN framework inherently employs hierarchical expert routing, it is uniquely positioned for deployment within the heterogeneous computational environments typical of modern Smart Grids. Moving forward, the practical implementation of this framework can greatly benefit from recent advances in resource-aware model collaboration, such as the large-small model collaborative paradigms recently demonstrated in heterogeneous edge networks [Reference to [47]. By leveraging this collaborative philosophy, the H-TLNN architecture can be optimally distributed: the Stage 1 Transformer module can reside on resource-abundant concentrator nodes to handle robust coarse classification, while the corresponding computationally efficient Stage 2 LNN experts can be dynamically deployed directly onto constrained edge meters. This synergistic approach promises to maximize both deployment efficiency and real-time inference capabilities across the grid’s disparate infrastructure.
